# Photodegradation of 2D Ruddlesden‐Popper Perovskites: Consequences and Design Principles for Photoelectrochemical Applications

**DOI:** 10.1002/advs.202507300

**Published:** 2025-07-18

**Authors:** Manuel F. Vasquez‐Montoya, Maxim Simmonds, Jinzhao Li, Anton Dzhong, Thomas W Gries, Arsene Chemin, Tristan Petit, Philippe Holzhey, Steve Albrecht, Sergei Trofimov, Boris Naydenov, Roel Van de Krol, Marco Favaro, Eva Unger

**Affiliations:** ^1^ Department of Solution‐Processing of Hybrid Materials and Devices Helmholtz‐Zentrum Berlin für Materialien und Energie GmbH Kekuléstraße 5 12489 Berlin Germany; ^2^ Department Novel Materials and Interfaces for Photovoltaic Solar Cells Helmholtz‐Zentrum Berlin für Materialien und Energie GmbH Kekuléstraße 5 12489 Berlin Germany; ^3^ Univ Lyon, Univ Claude Bernard Lyon 1, CNRS Institut Lumière Matière Villeurbanne F‐69622 France; ^4^ Young Investigator Group Nanoscale Solid‐Liquid Interfaces Helmholtz‐Zentrum Berlin für Materialien und Energie GmbH Albert‐Einstein‐Straße15 12489 Berlin Germany; ^5^ Department Perovskite Tandem solar Solar Cells Helmholtz‐Zentrum Berlin für Materialien und Energie GmbH Kekuléstraße 5 12489 Berlin Germany; ^6^ Fakultät IV – Elektrotechnik und Informatik Technische Universität Berlin 10587 Berlin Germany; ^7^ Department Spins in Energy Conversion and Quantum Information Science Helmholtz‐Zentrum Berlin für Materialien und Energie GmbH Albert‐Einstein‐Strasse 16 12489 Berlin Germany; ^8^ Institute for Solar Fuels Helmholtz‐Zentrum Berlin für Materialien und Energie GmbH Hahn‐Meitner‐Platz 1 14109 Berlin Germany

**Keywords:** 2D perovskites, PEA_2_PbI_4_, PEC, Photodegradation, photoelectrodes

## Abstract

Halide perovskites (HaP), with their exceptional optoelectronic properties and high‐power conversion efficiencies in photovoltaic devices, hold promise for photoelectrochemical (PEC) applications in green fuel and chemical production. However, their stability in aqueous environments remains a challenge. This study investigates the stability and degradation mechanisms of the 2D Ruddlesden‐Popper phase phenylethyl ammonium lead iodide (PEA^(+)^_2_PbI_4_) thin films in aqueous electrolytes under dark and illuminated conditions. While PEA^(+)^_2_PbI_4_ thin films appear to be thermodynamically stable in an aqueous electrolyte with phenylethyl ammonium iodide (PEAI), illumination causes significant photodegradation generating a deprotonated and dehalogenated 2D intercalation product: phenylethylamine‐lead iodide, 2PEA^(0)^‐PbI_2_. The degradation of the 2D semiconductor leads to substantial reduction in the photovoltage, adversely impacting the material performance in photoelectrochemical (PEC) devices. To intercept photo‐excited charge carriers in the 2D semiconductor, the I_3_^−^/I^−^ redox is added, which reduced photodegradation. The findings underscore that while catalytic reactions at halide perovskite electrodes in aqueous electrolytes are feasible, reversible and irreversible photodegradation remains a critical limitation that must be addressed in the design of PEC devices employing metal halide semiconductor layers for direct electrochemical energy conversion.

## Graphical abstract

In this study, we examined the structural and morphological stability of the 2D perovskite‐phenylethylammonium lead iodide PEA^(+)^_2_PbI_4_ thin films in aqueous electrolytes of phenylethylammonium iodide (PEA^(+)^I), both in the dark and under illumination. In dark conditions, the material remains stable due to dynamic equilibrium with the species in solution. However, under illumination, we demonstrate the formation of an intercalated phase 2PEA^(0)^−PbI_2_, which affects device performance. This work presents insightful design principles for utilizing 2D perovskite materials in photoelectrochemical applications.

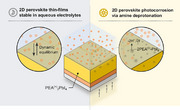

## 1 Introduction

Harvesting sunlight to drive technologically important electrochemical transformations at semiconductor/electrolyte interfaces envisages offers a promising avenue for producing green, affordable fuels and chemicals while addressing the intermittent nature of solar power.^[^ ^1^, ^2^, ^3^ ^]^ Despite decades of effort, the lack of sufficiently abundant, stable and intrinsically active semiconductor photoelectrode remains the long‐standing bottleneck toward scalable and viable photoelectrochemical (PEC) devices.^[^ ^4^, ^5^ ^]^ Metal halide semiconductors stand out due to their remarkable optoelectronic properties, tunable band gaps, and ease of fabrication, positioning them as potential game changers in the field of solar fuels, organic synthesis or environmental remediation.^[^ ^6^, ^7^, ^8^, ^9^ ^]^

However, the inherent instability of these semiconductors in protic electrolytes, especially in water, limits the viability of these materials in PEC devices and therefore the practical deployment of such technology.^[^ ^2^, ^10^ ^]^ To overcome this limitation, two main approaches have been implemented to prevent spontaneous dissolution of metal halide semiconductors (MHS) in aqueous environments: i) the use of protective layers to block electrolyte penetration (hence creating buried junctions).^[^ ^11^, ^12^, ^13^ ^]^ and ii) introducing constituent ions into the electrolyte, thereby preventing or minimizing dissolution by controlling the chemical equilibrium at the solid/liquid interface.^[^ ^6^, ^14^, ^15^ ^]^ The latter method is particularly relevant because it offers a versatile strategy for stabilizing the perovskite/electrolyte interface directly, without the need for complex and costly depositions of protecting/passivating overlayers.^[^ ^10^ ^]^

Studies on hydrogen iodide (HI) splitting at metal halide perovskite (MHP) semiconductor as particulate photocatalysis demonstrated the role of electrolyte manipulation for photoelectrochemical (PEC) applications. Pioneering research by Park et al.^[^ ^16^ ^]^ shown the stabilization of methylammonium lead iodide (MAPbI_3_) in concentrated hydroiodic acid (HI) aqueous solutions by establishing a dynamic dissolution‐reprecipitation equilibrium. Under illumination, the generation of hydrogen (H_2_) and an increment of triiodide ions (I_3_^−^) in solution was observed. These products were hypothesized to result from the photoelectrochemical splitting of HI. Subsequent work extended this approach to different 2D lead iodide hybrid perovskites. Wang et al.^[^ ^17^ ^]^ studied phenyl alkylammonium 2D Ruddlesden‐P‐opper (RP) perovskite‐derived absorbers as photocatalysts for the hydrogen evolution reaction, demonstrating superior efficiency compared to their 3D counterparts. Peng et al.^[^ ^18^ ^]^ studied aminoethyl pyridinium‐based Dion‐Jacobson (DJ) perovskite‐derived absorbers as photo‐electrochemically active material, also demonstrating hydrogen generation. Interestingly, these studies suggest that 2D perovskites can be stabilized by adding the organo‐halide cation into the electrolyte, removing the need to saturate the aqueous solutions with lead as in the case of the 3D counterparts. Complementing these significant findings, further research has focused on boosting the photon‐to‐hydrogen conversion efficiency through methods like co‐catalyst incorporation,^[^ ^19^, ^20^ ^]^ compositional adjustments,^[^ ^21^, ^22^, ^23^ ^]^ and the development of heterojunction structures.^[^ ^24^, ^25^, ^26^, ^27^ ^]^

However, the main challenge in developing PEC cells, especially in the context of MHS absorber materials, is their tendency to undergo light‐induced decomposition.^[^ ^4^, ^28^ ^]^ Photo‐redox processes and light‐induced degradation mechanisms have been widely investigated for metal‐halide perovskite used as absorber layers in thin‐film photovoltaics but have been largely unexplored in the context of photocatalysis applications.^[^ ^10^, ^29^, ^30^, ^31^ ^]^ Gaining a clear understanding of the conditions and mechanisms behind the photodegradation, along with multimodal methods to demonstrate its impact under direct operating conditions, is essential for developing stable and efficient MHS‐based PEC devices.

In this study, we examined the structural and morphological stability of the 2D MHP phenylethylammonium lead iodide PEA^(+)^_2_PbI_4_ thin‐films in aqueous electrolytes of phenylethylammonium iodide (PEA^(+)^I) both in the dark and under illumination. Note, that we distinguish between the protonated phenylethyl ammonium and the deprotonated phenylethylamine using the symbol (+) in the acronym, which is of utmost importance in the context of this study, as the reader will understand later. Our finding reveals that PEA^(+)^_2_PbI_4_ thin‐films are “stabilized” by excess of PEA^(+)^I aqueous electrolytes in the dark, but changes in the optoelectronic quality and morphology of the material suggest that the initially deposited thin‐films undergo chemical changes. Under illumination conditions (≈1 sun), the formation of a deprotonated and dehalogenated phenylethylamine‐lead iodide adduct (2PEA^(0)^‐PbI_2_) and localized material loss is observed, suggested to stem from the irreversible photodegradation of PEA^(+)^_2_PbI_4_. Consequently, we observed a strong decay in the open‐circuit potential (OCP) under illumination, limiting the device performance. This research underscores the need of distinguishing photocatalytic and photodegradation reactions on the molecular level to design “stable” or self‐regenerating systems facilitating a high solar‐to‐fuel conversion efficiency of perovskite‐based PEC devices, thereby advancing the development of sustainable solar fuel and chemical production technologies.

## 2 Results and Discussions

**Figure 1:**
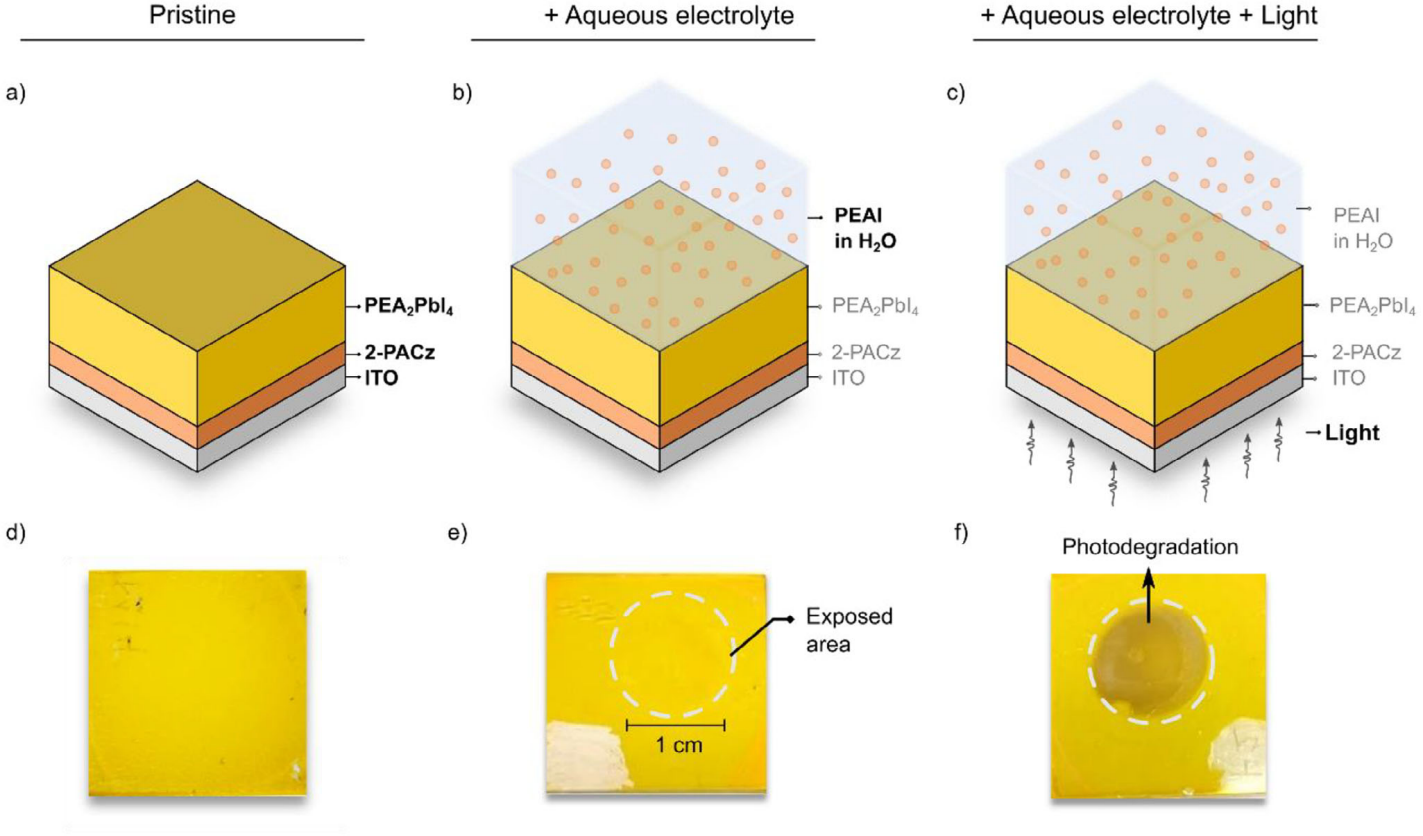
Experimental conditions and images of PEA_2_PbI_4_ thin films after exposure. Panels a–c) show the three experimental conditions: (a) pristine thin film on 2‐PACz hole‐selective layers on ITO, (b) thin film exposed to 0.3 m aqueous PEA^(+)^I electrolyte in the dark, and (c) thin film illuminated by a xenon lamp (≈1 sun) while immersed in the PEA^(+)^I electrolyte. Panels d–f) display the appearance of the thin films after these conditions: (d) pristine sample; (e) no visible changes are observed upon exposure to the aqueous electrolyte in the dark, demonstrating stability; (f) the area exposed to the electrolyte under illumination shows a color change, indicating photodegradation.

Thin films of the 2D Ruddlesden Popper (RP) perovskite‐derived absorber PEA^(+)^_2_PbI_4_ were deposited on conducting indium tin oxide (ITO) covered with a self‐assembled monolayer of hole‐selective 2‐PACz (2‐(9H‐carbazol‐9‐yl)ethyl)phosphonic acid) as depicted in **Figure** **1**a. The thickness of the film is ≈220 nm (see below). For the sake of clarity, the phenylethyl ammonium cation will be represented as PEA^(+)^, and the deprotonated phenylethylamine as PEA^(0)^. This study evaluates the stability of PEA^(+)^_2_PbI_4_ as photoelectrode, analyzing its chemical and morphological transformations in aqueous PEA^(+)^I under dark and illuminated conditions (Figures 1a–c).

As shown in Figure 1d,e, no discernible changes in the appearance of the film were observed following immersion in the electrolyte for 1 week in an aqueous solution 0.4 m PEA^(+)^I. Consistent with prior reports by Ma et. al.^[^ ^32^ ^]^ and Wang et. al.^[^ ^17^ ^]^ conducted on RP powders, the thin films remain stable in water at concentrations of PEA^(+)^I greater than 0.15 m, evident from Figure S1 (Supporting Information).

However, when exposed to light (10 min at ≈1 sun) while in contact with the electrolyte, the sample exhibits a significant change in appearance compared to the pristine sample (Figure 1f). This visible change is a direct indicator of photo‐induced chemical changes of the 2D absorber exposed to aqueous solutions, even in the presence of PEA^(+)^I, used to chemically stabilize the photoabsorber. In light of these qualitative observations, a more detailed analysis of structural, morphological, and chemical changes in the 2D perovskite absorber were carried out, which will be discussed in the following sections.

### 2.1 Structural characterization: Observation of 2PEA^(0)^‐PbI_2_ Degradation Product

**Figure 2:**
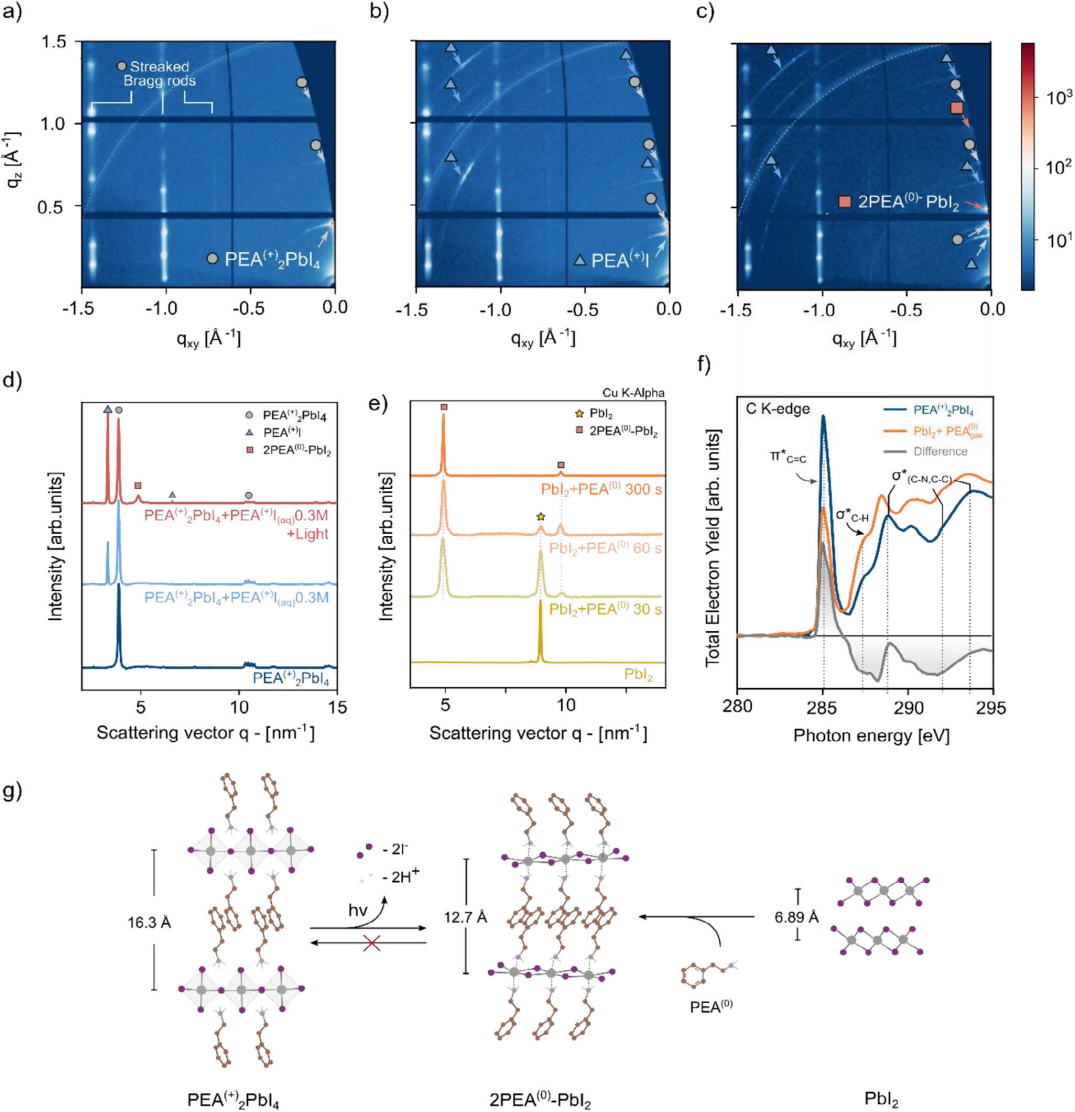
Structural changes in thin films under the three experimental conditions electrolyte immersion and illumination. Panels a–c) show 2D diffraction patterns taken at an angle of 0.3° with respect to the sample surface: (a) the pristine sample shows the PEA^(+)^_2_PbI_4_ composition with in‐plane oriented film, (b) immersion for 10 min in PEAI aqueous electrolyte reveals additional reflections attributed to electrolyte precipitation, and c) immersion in the electrolyte for 10 min under illumination conditions (≈1 Sun) leads to a new phase, attributed to 2PEA^(0)‐^PbI_2_. Panel d) displays the azimuthal integrated diffractogram for all three conditions. e) XRD diffractogram of lead iodide (PbI_2_) exposed to phenylethylamine gas (PEA^(0)^) at different times inside a glovebox. Resulting in the formation of the assigned 2PEA^(0)^‐PbI_2_ adduct. f) Near edge X‐ray absorption fine structure (NEXAFS) spectroscopy of the C‐K edge reveals a change on the molecular configuration between the pristine and adduct phase. g) Provide a graphical representation of the observed transformations. Upon illumination, PEA^(+)^_2_PbI_4_ transforms into 2PEA^(0)^‐PbI_2_, involving the loss of two equivalents of iodine and hydrogen from the pristine perovskite structure. The same 2PEA^(0)^‐PbI_2_ phase is obtained upon exposure of PbI_2_ to the phenylethylamine gas (PEA^(0)^).

Structural changes of the 2D PEA^(+)^_2_PbI_4_ perovskite were analyzed by comparing grazing incidence wide‐angle X‐ray scattering (GIWAXS) data of pristine samples in contrast with samples exposed to the electrolyte environment with or without illumination. **Figure** **2**a–c display the reciprocal 2D diffraction patterns for the pristine sample, the sample after contact with the electrolyte in the dark, and the same upon exposure to visible light, respectively. In Figure 2a, the pristine sample exhibits characteristic reflections of PEA^(+)^_2_PbI_4_ at q values of 3.8, 7.7, 11.5, and 15.4 nm⁻¹, associated with the (002), (004), (006) and (008) planes, respectively. Additionally, characteristic Bragg rods are observed. These reflections suggest a highly oriented in‐plane crystallization of the 2D perovskite.^[^ ^33^, ^34^, ^35^ ^]^ From the experimental data, the interlayer distance was estimated to be 1.63 ± 0.01 nm (see Figure S2a, Supporting Information) in agreement with previously reports.^[^ ^36^ ^]^ Upon exposure of the sample to the electrolyte, the 2D diffraction pattern shown in Figure 2b exhibits reflections consistent with the 2D PEA^(+)^_2_PbI_4_ absorber. However, we observe a small shift in the (002) plane peak toward lower q values (of the order of Δq = 0.04 nm⁻¹, Figure S3, Supporting Information), likely indicating a slight expansion of the crystallographic structure.^[^ ^37^ ^]^ Additional reflections observed at q values of 3.3, 6.6, and 13.1 nm⁻¹, and along the q_r_ value of 12 nm^−1^, are consistent with precipitated PEA^(+)^I which we attribute to residues deposited on the sample after immersion in the PEA^(+)^I saturated aqueous solution (see Figure S4, Supporting Information). Notably, we do not observe appreciable changes in the crystal structure of the PEA^(+)^_2_PbI_4_ thin films inside the aqueous electrolyte after 6 months of immersion in the electrolyte as shown in the Figure S5 (Supporting Information). It is worth noting that the observed stability does not extend to all RP cations; under identical conditions, films based on Butylammonium and Octylammonium undergo rapid dissolution (Figure S6, Supporting Information).

Upon exposing the sample to the electrolyte and light (Figure 2c), the formation of a new phase is observed with characteristic peaks at q values of 4.91 and 9.84 nm^−1^, in addition to the already mentioned PEA^(+)^_2_PbI_4_ and PEA^(+)^I reflections. These diffraction peaks are attributed to the formation of an adduct phase of phenylethylamine and lead iodide with a stoichiometry of 2:1 (2PEA^(0)^‐PbI_2_), which has been previously suggested by Kitazawa et al.^[^ ^38^, ^39^ ^]^ A lattice spacing of 1.27 ± 0.01 nm was determined for this phase from the experimental q values (See Figure S2b, Supporting Information).

As a reference sample, we synthesized the pure phenylethylamine intercalated lead iodide, 2PEA^(0)^‐PbI_2_, by exposing lead iodide, PbI_2_ thin films to phenylethylamine, PEA^(0)^ gas at different reaction times under inert atmosphere. Experimental X‐ray diffraction data (note that plots are respective the Cu‐Kα X‐ray source) of the synthesized reference compounds for different PEA‐gas exposure times are shown in Figure 2e. The sequential disappearance of the plane (002) of the PbI_2_ thin‐film and the appearance of two new peaks at lower q values of 4.91 and 9.84 nm^−1^ (6.90° and 13.86° in 2θ degrees for Cu‐ K alpha), suggest an intercalation process. After 300s of exposure to gas, only the peaks attributed to the adduct phase are observed, which is identical to the phase observed in GIWAXS of photo‐degraded PEA^(+)^_2_PbI_4_ samples.

To further characterize the interactions of the phenylethylamine molecules and gain insights into the molecular constitution in the adduct‐phase of 2PEA^(0)^‐PbI_2_, we performed near‐edge X‐ray absorption fine structure spectroscopy (NEXAFS). Figure 2f compares the carbon K‐edge transitions between the 2PEA^(0)^‐PbI_2_ adduct and the pristine sample PEA^(+)^_2_PbI_4_. The most intense transition, centered at 285.3 eV, is assigned to the $\pi_{C=C}^{*}$ resonance. The shoulder visible at 287.5 eV is assigned to $\sigma_{C-H}^{*}$ transitions, whereas the peaks series between 289 and 294 eV corresponds to $\sigma_{\left(C-N,C-C\right)}^{*}$ transitions. Finally, the broad feature centered at 303 eV is assigned to the $\sigma_{C=C}^{*}$ transition.^[^ ^40^, ^41^ ^]^ The main changes in the energetic position are observed in the $\sigma_{\left(C-N,C-C\right)}^{*}$ features, in agreement with the chemical differences between the ammonium and amine functional groups. The deprotonation of the amino groups is confirmed by the reduction of the $\sigma_{\left(N-H\right)}^{*}$ and $\sigma_{\left(C-N,\right)}^{*}$ transitions on the NEXAFS at the N K‐edge while no clear changes are observed at the I M‐edge (Figure S7, Supporting Information). In addition, the change in intensity on the $\pi_{C=C}^{*}$ transition suggest a different orientation of the phenylethylamine molecule on the adduct phase.^[^ ^42^ ^]^ Note that the NEXAFS investigation was performed in total electron yield (TEY), meaning that all the non‐radiative, electron‐mediated de‐excitation processes (i.e., primary, Auger, and secondary electrons) contribute to the signal. B. H. Frazer and co‐workers demonstrated, however, that the Auger electron contribution to TEY intensity is negligible.^[^ ^43^ ^]^ Hence, the probed depth of the measurement, however affected by the a degree of uncertainty given to the multiple signal source, spans from a few nm to a few tens of nm.^[^ ^43^ ^]^

From our results, we suggest that two concerted or consecutive processes occur under illumination: (1) the deprotonation of the phenylethyl ammonium to amine, formally:

(1) $$C_{6}H_{5}-{\left(CH_{2}\right)}_{2}-NH_{3}^{+}\to C_{6}H_{5}-{\left(CH_{2}\right)}_{2}-NH_{2}^{0}+H^{+}$$

And (2) the dehalogenation of the [PbI_4_]^−2^ to [PbI_2_]^(0)^, formally:

(2) $${\left[\mathrm{Pb}I_{4}\right]}^{2-}\to {\left[\mathrm{Pb}I_{2}\right]}^{\left(0\right)}+2I^{-}$$

Note that in the process, the 2D layer of corner‐sharing lead‐halide octahedra in PEA^(+)^_2_PbI_4_ stabilized by the PEA^(+)^ cations is converted into the amine 2PEA^(0)^‐PbI_2_ structure. In this PEA^(0)^‐intercalated PbI_2_ structure, the PbI_2_ units are edge‐sharing. The charge‐neutral amine PEA^(0)^ act as a Lewis‐base, likely interacting with the PbI_2_ layers via van‐der‐Waals forces similar to other reported haloplumbate(II) complexes like 2DMSO‐PbI_2_, Pyridine‐PbI_2_, or Aniline – PbI_2_.^[^ ^44^, ^45^, ^46^ ^]^ Interestingly, upon immersing the 2PEA^(0)^‐PbI_2_ adduct compound into the PEA^(+)^I electrolyte, we did not observe a conversion to PEA^(+)^_2_PbI_4_. This suggests that the deprotonation reaction is not reversible in the selected electrolyte media.

The observations are of particular importance for the alleged PEC activity of PEA^(+)^_2_PbI_4_ toward hydrogen generation via HI splitting. Importantly, our findings suggest that hydrogen and iodine may originate from the photoabsorber decomposition upon illumination, namely from the partial deprotonation of the ammonium group and the dehalogenation of the iodoplumbate(II) structure. Experimental results claiming catalytic HI splitting should hence be scrutinized as to this potential photo‐degradation mechanism being the actual source of H_2_ and I_2_.

### 2.2 Morphological Characterization: Localized Pin‐Hole Formation

To further understand the degradation processes observed in the GIWAXS analysis, we utilized constant amplitude non‐contact mode atomic force microscopy (AFM) combined with frequency modulation (FM) sideband Kelvin‐probe force microscopy (KPFM) and confocal laser scanning microscopy (CLSM) to analyze the post‐exposure samples. This approach allowed us to investigate the morphological changes in thin films subjected to different experimental conditions, providing deeper insights into the morphological changes upon immersion in the electrolyte.

**Figure 3:**
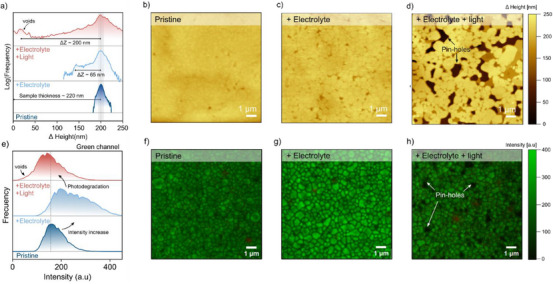
Morphological changes on the thin films at the three experimental conditions measured by atomic force microscopy (AFM) and confocal laser scanning microscopy (CLSM). a) Histogram distribution of heights, representing the different changes in depth after electrolyte and illumination conditions. b) Pristine sample presents a relatively flat, homogeneous height profile. c) Electrolyte immersion causes restructuring in the dark, forming voids ≈65 nm deep d) Exposure to the combined action of electrolyte and light results in severe structural damage, with void depths extending to the entire film thickness. e) Histogram distribution of the green channel (502 ≤ λ ≤ 546) representing the changes in photoluminescence after the experimental conditions. f,g,h) Maximum intensity projection of a Z‐scan with a CLSM of the Pristine, electrolyte, electrolyte and light samples. Zoomed height image reveals distinct height variations in the profile distribution of the photodegraded sample.

**Figure** **3**a shows the histogram of the AFM profile of the three conditions. The pristine sample (Figure 3b) exhibits a flat morphological profile with barely visible grain boundaries. Significant changes are observed in the morphological profile after exposure of the sample to the electrolyte in the dark. As shown in Figure 3c, the interaction of the sample with the aqueous electrolyte results in modifications of the microstructure, with more defined grain boundaries and accompanied with the appearance of voids. These voids have a depth of ≈65 nm, which do not exceed the thickness of the 2D thin film. The grain domains become more evident with increased contrast in morphological profile depth, suggesting an Ostwald ripening effect.^[^ ^47^ ^]^ or grain etching.^[^ ^48^ ^]^ The appearance of small structures on top of the grain boundaries are attributed to 2D perovskite recrystallized after drying the sample.

When exposing the sample to the combined effect of electrolyte and light (Figure 3d), the sample morphology changes significantly: grains become sparser with larger dark areas indicating voids. As a result, differences in the height profile become significantly more prominent. The height difference between the surface and dark voids is on the order of 220 nm, indicating that voids extend through the entire thickness of the 2D perovskite film. The distribution of these voids over the sample surface reflects another clear sign of photodegradation.

To address the impact of the change in morphology on the optoelectronic properties of the material, we measured confocal laser scanning microscopy (CLSM) as shown in Figure 3e–h. Figure 3e represent the histogram variation on intensity comparing the samples exposed to the three different conditions monitoring the green channel (502 **≤ λ ≤** 546 nm), attributed to the main photoluminescence (PL) signal for PEA_2_PbI_4_. After immersion in the electrolyte (Figure 3g), the strongly enhanced PL signal suggests an improvement in radiative recombination. Interestingly this improvement does not follow a normal distribution as represented in Figure 3e (Middle section – Electrolyte condition). After illumination (Figure 3h), a clear reduction in the PL intensity is observed across the sample, especially in localized areas assigned to the voids formation, in complement with the AFM measurements. Additionally, we detected emission in the red channel (666 **≤ λ ≤** 732 nm), assigned to localized material defects with a broad sub‐bandgap emission.^[^ ^49^ ^]^ Despite being present also in the pristine sample, the signal is further enhanced in the presence of illumination, suggesting an increase in localized defects after the exposure of the combined effect of electrolyte and illumination (Figures S8 and S9, Supporting Information).

Another significant change is observed in the sample work function (WF) for the three investigated conditions (Figure S10, Supporting Information). After immersion of the electrolyte, the pristine sample with a homogeneous WF across the surface ((4.48 ± 0.04) eV) transforms into two distinct domains: grains with a WF of (4.65 ± 0.07) eV and grain boundaries with a WF of (4.52 ± 0.04) eV. Interestingly, after illumination the WF values of grains and grain boundaries shift to lower values, (4.51 ± 0.05) eV and (4.37 ± 0.05) eV, respectively. However, the relative change in WF between the grains and the grain boundaries remains constant after illumination, with a difference of 0.15 eV. This consistency is maintained despite the different height profiles observed in the AFM morphology. We suggest that the change in WF across the sample is attributed to the local formation of the 2PEA^(0)^‐PbI_2_ adduct on the surface of the photoabsorber.

To gain further insight into the spatial effects on photodegradation, we performed in situ PL measurements on PEA₂PbI₄ crystals under continuous illumination (Figure S11, Supporting Information). Six distinct regions were monitored, with zones 1 and 2 located at the crystal edges and the remaining zones within the interior. A pronounced decrease in PL intensity was observed at the edges (≈70% relative change in intensity), supporting their role as degradation initiation points. However, a similarly rapid drop in intensity within the first 50 s was also detected in the interior regions of the crystal. These observations suggest that photodegradation occurs along the material surface and not solely at the grain boundaries.

These experimental observations suggest that 1) immersion of the 2D perovskite sample into the aqueous electrolyte leads to a beneficial improvement of the optoelectronic quality of the material, presumably from the dynamic reconstruction in the electrolyte media, 2) after illumination, pinhole formation and a decrease in the optoelectronic quality of the sample is indicative of photodegradation of the samples. 3) the mechanism of degradation cannot be explained merely by the decomposition on the grain boundaries but rather by generalized damage occurring at the surface and near‐surface region of the material.

### 2.3 Spectro Electrochemical Characterization: Photovoltage Drop Over Time

**Figure 4:**
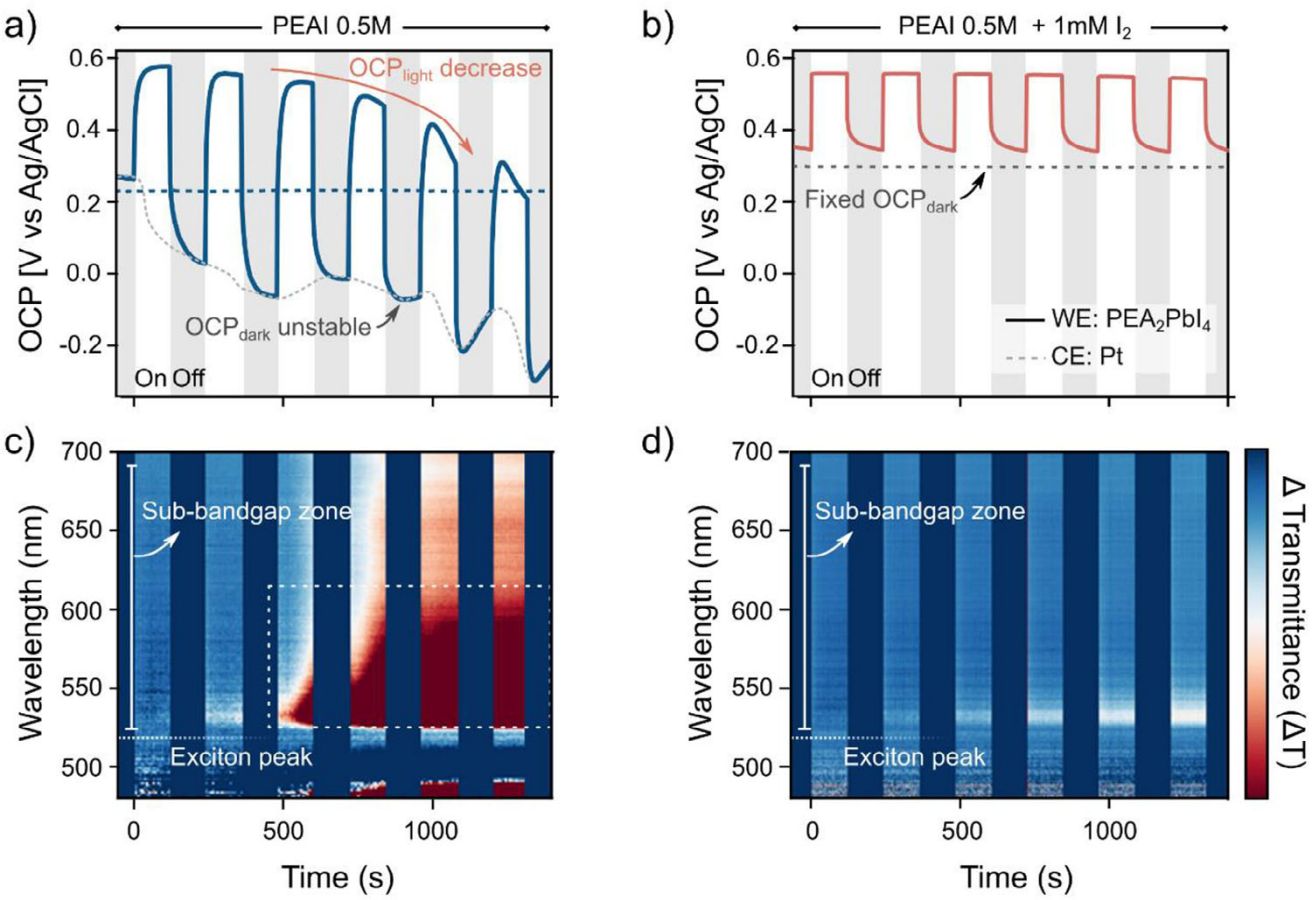
Photovoltage changes of the 2D perovskite thin film using a customized spectroelectrochemical setup with and without the I^−^/I_3_^−^ redox couple. a) Light‐chopped open‐circuit potential (OCP) measurements, showing an overall voltage decrease due to degradation and unstable dark potential. However, under the presence of the iodine redox couple b), the potential is greatly stabilized in dark and under illumination. Transmittance change (ΔT) heatmaps c) without and d) with iodine redox couple.

To further investigate the dynamics of the photodegradation process and gain a deeper understanding of the electrochemical behavior of the electrified solid/liquid junction, we transitioned from ex situ measurements to in situ spectroelectrochemical methods (**Figure** **4**). Here, we used a customized spectroelectrochemical experimental setup combining open‐circuit potential (OCP) measurements with in situ transmittance spectroscopy (see methods for details). Using this approach, it is possible to link the changes to the electronic properties of the samples immersed in electrolyte solutions with optical changes in the material, indicating material dissolution or surface coarsening and thereby providing direct information of photodegradation processes.^[^ ^10^ ^]^ The results are shown in Figure 4.

To accurately interpret this response, it is necessary to address the variations both in the dark and under illumination. In the dark, the OCP is governed by the redox potential of the solution defined by the Nernst equation.^[^ ^50^ ^]^ of the reduced and oxidized species present in the electrolyte. In the PEA^(+)^I electrolyte, only the reduced species (I^−^) are present in the solution. This point is crucial since measurements in the absence of redox mediators yield different responses in the dark (see Figure 4a), most likely due to the presence of mixed potentials according to the Wagner‐Traud additivity principle of half‐reaction potentials,^[^ ^51^, ^52^ ^]^ thus complicating the interpretation of the PEC data. To overcome this issue, we added 1 mm of I_2_ to from an I^−^/I_3_^−^ redox mediator, defining the semiconductor/electrolyte junction potential,^[^ ^53^, ^54^, ^55^ ^]^ which is a redox couple widely used in dye‐sensitized solar cells (DSSCs) due to the fast kinetics of its inner‐sphere electron transfer.^[^ ^56^ ^]^ The OCP measured in the dark with and without the I^−^/I_3_^−^ redox couple was (+0.233 ± 0.005) V and (+0.298 ± 0.005) V versus Ag/AgCl/Cl^−^_(sat.)_, respectively. Under illumination, we observe a positive shift of the OCP (*OCP_light_*) relative to the *OCP_dark_*, reaching a maximum value of 0.57 V versus Ag/AgCl/Cl^−^_(sat.)_ with and without the redox couple. The *OCP_light_* was observed to decrease over the course of the dark/light cycling more pronounced in the situation without the redox mediator.

The positive shift under illumination relative to the dark equilibrium potential indicates that holes are being extracted toward the self‐assembled monolayer (2PACz) interface (the back contact), which is consistent with the expected behavior of a hole‐selective layer (HSL). This observation is supported by Figure S12 (Supporting Information), which shows a negative shift in the OCP upon illumination when using TiO_2_, a well‐known electron selective contact (ESL).^[^ ^57^ ^]^ Similar to other excitonic PEC solar devices, such as dye‐sensitized solar cells, quantum dots, or organic solar cells, the presence of a selective contact layer dictates the sign of the photovoltage and determines the magnitude of the photovoltage upon illumination based on carrier selectivity.^[^ ^58^, ^59^ ^]^ The OCP values measured are close to the work function of the 2PACz/ITO interface,^[^ ^60^ ^]^ suggesting that the photovoltage is pinned by the HSL layer.

To unveil the relationship between photovoltage changes and sample degradation, we analyzed the differences in the optical transmission spectra during the illumination cycles, represented in Figure 4c,d. We observe that changes occur mostly below the bandgap onset (non‐absorbing part), ≈530 nm. These spectral changes are attributed to increased light scattering, suggesting changes in the sample roughness as observed in the AFM measurements. Confirming of this are transmission and reflection spectra, shown in Figure S13 (Supporting Information). They show that the changes in the non‐absorbing region primarily originate from the reflection spectra rather than the transmission spectra. However, the exciton peak located at 520 nm does not change during the illumination period (Figure S14, Supporting Information) indicating that changes in the sample absorbance are instead negligible in this spectral range due to the strong absorption of the excitonic transition.

Ex situ GIWAXS and AFM measurements with and without the redox mediator are represented in Figure S15 (Supporting Information). Notably, the sample immersed in the solution containing the I^−^/I_3_^−^ redox couple does not show the distinctive features of the intercalated phase. From these experimental results we infer that in the presence of the I^−^/I_3_^−^ redox couple, the formation of the 2PEA^(0)^‐PbI_2_ adduct is inhibited. We hypothesize that the photodegradation of PEA^(+)^_2_PbI_4_ is prevented when photo‐excited charges are efficiently quenched by the redox couple. This interpretation is supported by experimental data on the sample microstructure (Figure S15b,c, Supporting Information), where the material morphology decomposition is strongly suppressed in the presence of the I^−^/I_3_^−^ redox couple. Therefore, we conclude that the dynamic changes observed at OCP, together with the modifications in the sample transmission, are correlated with the formation of the 2PEA^(0)^‐PbI_2_ photodegradation product and the consequent morphological reconstruction of the sample.

Notably, the presence of other redox mediators can strongly modify the photodegradation pathway. Figure S16 (Supporting Information) shows the OCP and transmission spectra changes in presence of two commonly‐used redox mediators, hexacyanoferrate (II)/(III) ([Fe^II/III^(CN)_6_]^4−/3−^) and hexaammineruthenium (II)/(III) ([Ru^II/III^(NH_3_)_6_]^2+/3+^). Here, the degradation kinetics are strongly modified compared to the I^−^/I_3_^−^, being more pronounced in the [Fe^II/III^(CN)_6_]^4−/3−^ couple than the [Ru^II/III^(NH_3_)_6_]^2+/3+^ redox couple. AFM and GIWAXS measurements taken after exposing the samples to these redox couples are reported in Figures S17 and S18 (Supporting Information).

The minimal degradation observed when the I^−^/I_3_^−^ redox mediator was added to the electrolyte is particularly interesting, especially given its similar redox potential to [Fe^II/III^(CN)_6_]^4−/3−^. Despite this similarity, striking differences in photodegradation are observed. This suggests that charge transfer occurring at the I^−^/I_3_^−^ mediator is influenced by a more intimate chemical interaction between the redox couple and the photoabsorber. I^−^/I_3_^−^ species likely either adsorbed on the material surface or even intercalate into the 2D materials, hence facilitating fast electron transfers. Similar observations have been reported for chalcogenides such as MoS_2_.^[^ ^53^, ^61^ ^]^ Density functional theory (DFT) calculations have shown that I_2_ molecules and interstitial I^−^ moieties can be energetically stabilized on the surface of halide perovskite materials,^[^ ^62^, ^63^ ^]^ supporting this hypothesis. Another important consideration is that introducing excess iodine (I_2_) into the solution can result in its interaction with defect states of the material, thereby modifying its electronic properties.^[^ ^64^ ^]^ These findings indicate that the redox potential alone does not determine the stability of the devices; the presence of surface states (and their energetics) plays a crucial role in this dynamic. While the exact mechanism of these interactions is beyond the scope of this article, our results underscore the importance of controlling the electrochemical environment to design effective and stable photoelectrodes for photoelectrochemical energy conversion.

## 3 Conclusion and Outlook

In this study, we investigated the photodegradation dynamics of PEA^(+)^_2_PbI_4_ thin films in aqueous electrolytes and their implications for photoelectrochemical (PEC) applications. Our key findings reveal that while PEA^(+)^_2_PbI_4_ thin films can be stabilized in an aqueous PEA^(+)^I electrolyte, they degrade under light irradiation. *Post mortem* analyses revealed significant morphological transformations and the formation of a deprotonated and dehalogenated 2PEA^(0)^‐PbI_2_ adduct. The photodegradation leads to a substantial reduction in photovoltage, adversely affecting the potential performance of PEC devices. The introduction of redox mediators reduces the rate of photodegradation. This effect is most pronounced for the I^−^/I_3_^−^ redox couple, indicating that apart from the extraction of photo‐excited carriers, the chemical interactions between the redox couple and the photogenerated carriers need to be considered. While further experiments are necessary to elucidate the exact chemical pathways for degradation, our study highlights the critical need to consider photo‐degradation reactions in the design of 2D halide perovskite photoelectrodes.

Based on the results discussed herein, we highlight the trade‐off condition imposed by stabilizing the PEA^(+)^_2_PbI_4_ thin films using PEA^(+)^I electrolytes for photoelectrochemical applications. While the solution can regenerate and enhance the material optoelectronic properties by a dynamic construction‐reconstruction process, the permeable interface also facilitates the material loss from the lattice, named deprotonation or dehalogenation, causing irreversible degradation on the thin‐film photoelectrode. Identifying operating conditions that promote material reformation over dissolution is crucial for designing durable photoelectrodes based on 2D RP halide materials.

To improve stability, more studies are required on the impact of the A‐site cation in the photodegradation, especially in the chemical and structural nature of the identified adduct PEA^(0)^‐PbI_2_ phase. Recent work by Ren et al,^[^ ^31^ ^]^ demostrates that deprotonation also occurs in other RP perovskites employing alternative cations such as 4‐fluorophenylethylammonium (F‐PEA), 2‐thiophenemethylammonium (TMA), and hexylammonium (HA). Notably, their findings indicate that Dion–Jacobson (DJ) perovskites exhibit slower degradation under illumination compared to their RP counterparts, highlighting the critical influence of the A‐site cation on material stability.

In parallel, modifying the halide composition could further enhance stability; for example, bromide‐based perovskites may exhibit greater resistance to photodegradation due to the stronger lead‐halide bond.^[^ ^34^ ^]^ However, such modifications must be carefully balanced against their impact on the material optoelectronic properties to ensure their viability for photoelectrochemical applications. In addition, further optimization on the device design such as interface passivation,^[^ ^48^ ^]^ crystallization orientation,^[^ ^34^, ^65^ ^]^ improvement on the selective transport layers^[^ ^66^ ^]^ and better alignment with the interested redox reaction,^[^ ^67^, ^68^ ^]^ can lead to beneficial improvement on the stability and efficiency of 2D perovskite as photoelectrodes.

Our findings underscore the critical importance of elucidating the photodegradation mechanisms in 2D perovskites, which is essential to reach a reliable application in solar energy conversion technologies. It is paramount to understanding the specific properties that initiate and propagate degradation under illumination. Here, advancements in characterization techniques and material engineering are required to systematically address these degradation pathways, especially on the understanding of how the electrochemical environment affects the decomposition reactions. By rigorously defining identifying these mechanisms, we can develop targeted strategies to mitigate degradation, significantly advancing the viability of 2D perovskites to drive photoelectrochemical reactions.

## Conflict of Interest

The authors declare no conflict of interest.

## Supporting information



Supporting Information

## Data Availability

The data that support the findings of this study are available from the corresponding author upon reasonable request.
