# Supplementary material for: Photodegradation of 2D Ruddlesden‐Popper Perovskites: Consequences and Design Principles for Photoelectrochemical Applications
Source: Adv Sci (Weinh). 2025 Jul 18;12(36):e07300. doi: 10.1002/advs.202507300 (PMC12463078; doi:10.1002/advs.202507300)
Supplement: Supplementary file 1 — Supporting Information [file ADVS-12-e07300-s001.docx]

Supporting Information

**Photodegradation in** **2D Ruddlesden-Popper Perovskites: Consequences and Design Principles for** **Photoelectrochemical Applications**

Manuel F. Vasquez-Montoya* ^1,8^, Maxim Simmonds^1^, Jinzhao Li^1^, Anton Dzhong^1^, Thomas W Gries^2^, Arsene Chemin^3,4^, Tristan Petit^4^, Philippe Holzhey^5^, Steve Albrecht^5,6^, Sergei Trofimov^7^, Boris Nayden^7^, Roel Van de Krol^8^, Marco Favaro*^8^, Eva Unger*^1^

*E-mail: manuel.vasquez@helmholtz-berlin.de, marco.favaro@helmholtz-berlin.de , eva.unger@helmholtz-berlin.de*

1. Department of Solution-Processing of Hybrid Materials and Devices, Helmholtz-Zentrum Berlin für Materialien und Energie GmbH, Kekuléstraße 5, 12489 Berlin, Germany

2. Department Novel Materials and Interfaces for Photovoltaic Solar Cells, Helmholtz-Zentrum Berlin für Materialien und Energie GmbH, Kekuléstraße 5, 12489 Berlin, Germany

3. Univ Lyon, Univ Claude Bernard Lyon 1, CNRS, Institut Lumière Matière, F-69622, Villeurbanne, France.

4. Young Investigator Group Nanoscale Solid-Liquid Interfaces, Helmholtz-Zentrum Berlin für Materialien und Energie GmbH, Albert-Einstein-Straße15, 12489 Berlin, Germany

5. Department Perovskite Tandem solar Solar Cells, Helmholtz-Zentrum Berlin für Materialien und Energie GmbH, Kekuléstraße 5, 12489 Berlin, Germany

6. Fakultät IV – Elektrotechnik und Informatik, Technische Universität Berlin, Berlin, 10587, Germany.

7.Department Spins in Energy Conversion and Quantum Information Science, Helmholtz-Zentrum Berlin für Materialien und Energie GmbH, Albert-Einstein-Strasse 16, 12489 Berlin, Germany

8. Institute for Solar Fuels, Helmholtz-Zentrum Berlin für Materialien und Energie GmbH, Hahn-Meitner-Platz 1, 14109 Berlin, Germany

Materials and Methods

*Materials*

Anhydrous dimethylformamide (DMF), phenylethylammonium iodide (PEAI), titanium diisopropoxide bis(acetylacetonate) Ti(acac)2(OiPr)2), hexaammineruthenium(II) chloride ([Ru(NH_3_)_6_Cl_2_]), hexaammineruthenium(III) chloride ([Ru(NH_3_)_6_Cl_3_] , Potassium hexacyanoferrate(II) trihydrate [K_4_Fe(CN)_6_] , Potassium hexacyanoferrate(III) [K_3_Fe(CN)_6_] and iodide crystals (I_2_, 99.999%) were purchased from Sigma Aldrich. Lead iodide (PbI_2_) and 2-(9H-carbazol-9-yl)ethyl)phosphonic acid (2PACz) were bought from TCI. Ethanol anhydrous was bought from VWR. All chemicals were used as received without further purification.

*2-PACz Hole selective layer preparation:*

Indium tin oxide (ITO) glass substrates were cleaned sequentially for 15 min with a 2% Mucasol (Schülke) solution in water, water, acetone, and isopropanol at ~ 40 °C in an ultrasonic bath. After that, directly before hole-selective contact deposition, the substrates were treated in an ultraviolet-ozone cleaner for 15 min. All subsequent procedures were done in a nitrogen-filled glovebox (MBRAUN).

For the hole-selective layer, 2PACz powder were dissolved in anhydrous ethanol at a concentration of 0.3 mg mL^–1^ and put into an ultrasonic bath for 15 min (30-40 °C) before using. The 2PACz coating was prepared by spin-coating of 100 μL of the solution, uniformly released onto the center of the substrate, the lid was closed and after ~ 5 s resting, the spin-coating program (30 s at 3000 rpm) was started. After spin-coating, the substrates were heated at 100 °C for 10 min.

*TiO_2_ Thin Film Preparation:*

For the compact TiO_2_ film (electron-selective contact), the cleaned substrates were treated with ultraviolet ozone (UV/O_3_, FHR UVOH 150 Lab) for 25 min at an oxygen flow rate of 1.0 L min^–1^. For the spray pyrolysis process, 150 µL of Ti(acac)_2_(O*^i^*Pr)_2_ were added to 15 mL of ethanol to yield a 20.2 mM precursor solution. The precursor solution was transferred to a reservoir vial equipped with the spray nozzle. Oxygen was used as a carrier gas during the process. The UV/O_3_-treated substrates were transferred to a high-temperature hot plate and heated to 450 °C. After stabilization for 10 min at 450 °C, the process was started. During the process, spraying intervals of 10 s duration were alternated with evaporation intervals of 30 s duration. The substates were then maintained at 450 °C for 1 h before the temperature was downregulated to 150 °C. The TiO_2_-coated substrates were used within 10 h for subsequent depositions or stored. In case of storage, the substrates were re-annealed at 450 °C for 1 h before subsequent steps [1].

*PEA_2_PbI_4_ Thin Film Preparation:*

For the PEA_2_PbI_4_ deposition, PbI₂ (345 mg) and PEAI (375 mg) were dissolved in DMF (2 mL) and stirred at room temperature for at least 2 h until fully dissolved, yielding a concentration of 0.37 M. The perovskite coating was prepared by spin-coating 100 μL of the solution, uniformly released onto the center of the substrate immediately after starting the spin-coating program. The spin-coating program involved two steps: 10 s at 1000 rpm followed by 60 s at 6000 rpm. After spin-coating, the substrates were annealed at 120 °C for 10 min.

*Electrolyte Preparation:*

Phenylethylammonium iodide (PEAI) solution was prepared with a concentration of 125 mg mL^–1^ in Milli-Q^®^ water (Resisitvity = 18.2 MΩ). The pH-value of the solution was measured as 6.82. The [Fe^II/III^(CN)_6_]^4–/3–^ solutions were prepared by dissolving 1 mM of potassium hexacyanoferrate(II) trihydrate and 1 mM of potassium hexacyanoferrate(III) in the PEAI solution. The [Ru^II/III^(NH_3_)_6_]^2+/3+^ solution was prepared by dissolving 1 mM of ruthenium hexaammineruthenium(II) chloride and 1 mM of hexaammineruthenium(III) chloride in the PEAI solution. For the I^–^/I_3_^–^ solution, 2 mM of I_2_ crystals in the prepared PEAI solution. All the samples were prepared fresh before every experiment, this is particularly important for the ruthenium derived probe, since overtime, some precipitation and color change in the solution is observed.

*PEA_2_PbI_4_ crystal growth:*

PEA₂PbI₄ crystals were synthesized following a previously reported method [2]. Briefly, PbI₂ and PEAI were dissolved in γ-butyrolactone (GBL) in a 1:2 molar ratio at 80 °C under continuous stirring overnight to yield a 2.12 M precursor solution, which was subsequently filtered. A drop of the solution was deposited onto a preheated substrate (float glass), covered with a second slide, and thermally annealed at 80 °C. Controlled crystallization was achieved by gradually cooling the assembly from 80 °C to 30 °C at a rate of 1 °C h⁻¹.

***Characterization Methods***

*X-ray characterization*

Grazing-incidence wide-angle X-ray scattering (GIWAXS) data were acquired at the myspot beamline of the synchrotron radiation facility BESSY II. Under air conditions, X-rays with 9 keV photon energy (*λ* = 1.378 Å) were incident on samples with a beam size of 50 x 50 µm. Grazing incidence angles scan from 0.1° to 2.0° within 20 steps were used to probe different depths in the film. In addition, lateral scan at 0.3° was performed every 0.6 mm to account different areas of the sample. All the measurements were performed with an integration time of 30 s. Scattering was detected with Dectrix Eiger X 9M detector (3000 x 3000 pixel, 75 µm pixel size). The beam center and SDD value of the GIWAXS reciprocal space images was calibrated with a standard LaB_6_ sample. Data was reduced and corrected using the software PyFAI[3].

*Near-edge X-ray absorption fine spectroscopy (NEXAFS)*

NEXAFS measurements were carried out at the HE-SMG beamline of the BESSY-II electron storage ring at Helmholtz-Zentrum Berlin (Germany), specifically optimized for NEXAFS spectroscopy in the VUV range, using an ultrahigh vacuum experimental station.[4] Data were collected in electron yield (EY) mode by sweeping the incident photon energy while simultaneously recording the emitted electrons from the sample under a 30 V screening. To minimize carbon contamination from the optical components, oxygen plasma cleaning was employed on all optics. Spectra were normalized between the pre-edge and post-edge regions for comparison without requiring background correction. The monochromator provided an energy resolution of approximately 70 meV around the C 1s X-ray absorption edge (~285 eV). The X-ray energy calibration was performed using the first narrow peak of the C 1s absorption spectrum of highly ordered pyrolytic graphite (HOPG) at ~285.45 eV.

*Atomic-force microscopy (AFM) Measurements*

The atomic force microscopy (AFM) measurements were conducted on a Park Systems NX12 AFM setup in air at room temperature. Topography of the samples was measured in a non-contact mode (NCM) using 160AC-NA (OPUS by MikroMasch) probe. Contact potential difference (CPD) spatial maps were obtained using conductive PPP-EFM (Nanosensors) cantilevers in a frequency modulated (FM) sideband KPFM mode [5], [6] at a modulation frequency of 3 kHz and an alternating current (AC) tip bias of 1 V. Work function (*ϕ*) spatial maps were derived from CPD maps according to the formula *ϕ* = *ϕ_tip_* − e CPD, where *e* is the elementary charge and *ϕ_tip_* is the work function of the tip, measured using freshly cleaved highly oriented pyrolithic graphite (HOPG) as a reference sample. In these experiments, *ϕ_HOPG_* was assumed to be 4.6 eV[7], [8].

Work=4.364 – e -0.10

Tip= 0.236 – 4.6

*Confocal Laser scanning microscopy:*

Confocal laser scanning microscopy (CLSM) was performed using a Nikon Eclipse Ti microscope equipped with a Nikon 100× 1.4 NA Plan Apo VC oil objective and a Nikon Spatial Array Detector (NSPARC). Excitation was done with a 405 nm LED, and exposure and laser power were equal for all images. The images are composed of two colours, collected with filters selectively collecting emission only from 502 to 546 nm wavelength range (green) and 666 to 732 nm (red). The sample was mounted in ambient air and a drop of immersion oil was placed on the objective. All samples were immediately measured after mounting. Please note that each channel (red and green) is scaled individually but the scale of each channel is the same for all images. So, for example, red has the same scale in all CLSM images. The green channel is two orders of magnitude more intensive than the red channel. The images are the maximum intensity projections of a depth scan with 0.1 μm stepwidth, which goes through the whole film with 0.1 μm stepwidth.

*Hyperspectral photoluminescence imaging*

The hyperspectral imaging was done with a HERA VNIR camera from NIREOS. The camera was mounted on a Nikon Eclipse Ti microscope with a Nikon 100× 1.4 NA Plan Apo VC oil objective. The excitation was done with a 405 nm LED. The same samples and similar areas were measured as also for the CLSM images.

*In-situ photoluminescence imaging*

In situ photoluminescence (PL) imaging was performed using a Thorlabs 415 nm LED as the excitation source. The excitation light was directed onto the sample through a 445 nm dichroic mirror, which also served to separate the excitation and emission pathways. The emitted PL signal was collected and recorded using a CS165MU compact monochrome camera (Thorlabs).

*Photoelectrochemical Characterization:*

The experiments were carried out using a home-build in-situ (photo)electrochemical flow cell. Digital photograph of the used cell and station is shown in figure S18. The liquid electrolyte is pumped from an external reservoir to the cell mounted in the analysis chamber via a liquid feedthrough (PEEK, 1/16 inch OD, 0.75 mm ID) using a 8-rotor peristaltic pump (Carl Roth, Cyclo II EP76.1), allowing the possibility to flow and recirculate the electrolyte. For all the experiments, the flow was kept constant at a flow speed of 1 ml min^–1^. The presence of the fluid capacitor enables the substantial suppression of the flow pulsation (due to the positive displacement pump). Prior to its introduction in the in situ (photo-) electrochemical cells, the electrolyte solution purged for at least 30 min with N_2_ gas at constant bubbling. To avoid the changes in a concentration, a bottle with milliQ water is bubbled to humidify the inlet of N_2_ gas. Before every experiment, the flow cell was flushed with MQ water to remove the electrolyte and sequentially dry it out using a nitrogen gun. Note that is important to ensure that the flow cell is completely dry to avoid degradation of the 2D perovskite in the assembly process.

For the electrochemical characterization, open-circuit voltage (*V_OC_*) measurements were conducted using a potentiostat/galvanostat (VSP-300, Biologic) with a simultaneous record of the potential at the working electrode (PEA_2_PbI_4_ device stack) and counter electrode (Pt). Flow cell compatible Ag/AgCl (saturated KCl) reference electrode was used inside the flow photoelectrochemical cell. Before each measurement, the reference electrode was measured against a master electrode Ag/AgCl (saturated KCl) electrode (XR300, Radiometer Analytical) in saturated KCl to ensure a stable operation.

The illumination of the sample was performed using a Xenon lamp (Muller Electronik Optic – Typ LAX) and collimated using aspherical lenses to a spot size of 5 cm. Neutral density filters (ND) where used to adjust the irradiance in the sample. If otherwise stated, the measurements were conducted using an 0.5 ND filter, giving an integrated irradiance of 1.09 x 10^3^ W m^–2^. The intensity was calibrated using a calibrated spectrometer (ocean optics 2000+). The spectra is illustrated in Figure S20. All measurements were carried out in a back-side illumination configuration (light incident through the ITO side).

For the in-situ transmission measurements, a CCD spectrometer (Thorlabs, CCS200) coupled with a cosine corrector was placed in free-space configuration at 1 cm of the photoelectrochemical cell. The integration time was set at 5 ms with an average of 1000 counts. The data was acquired using a self-written Python program.


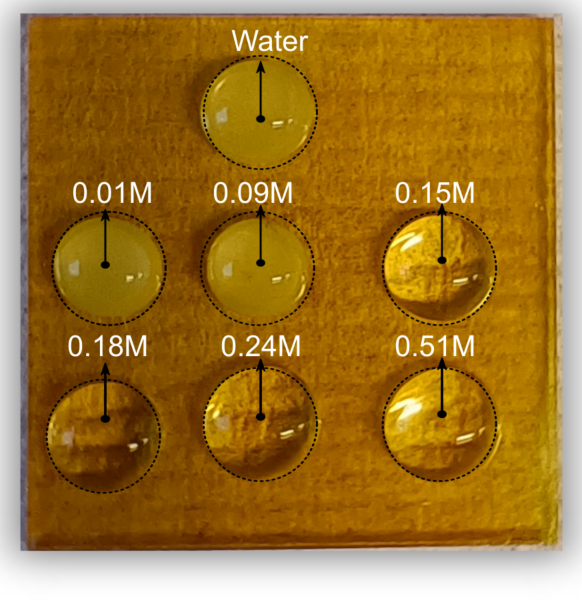


**Figure S1:** Concentration series of PEA^(+)^I^-^ aqueous solutions to validate the required concentration to stabilize the thin-films in the electrolyte solution. As can be observed, stability of the thin films is reached at concentrations higher than 0.15M.


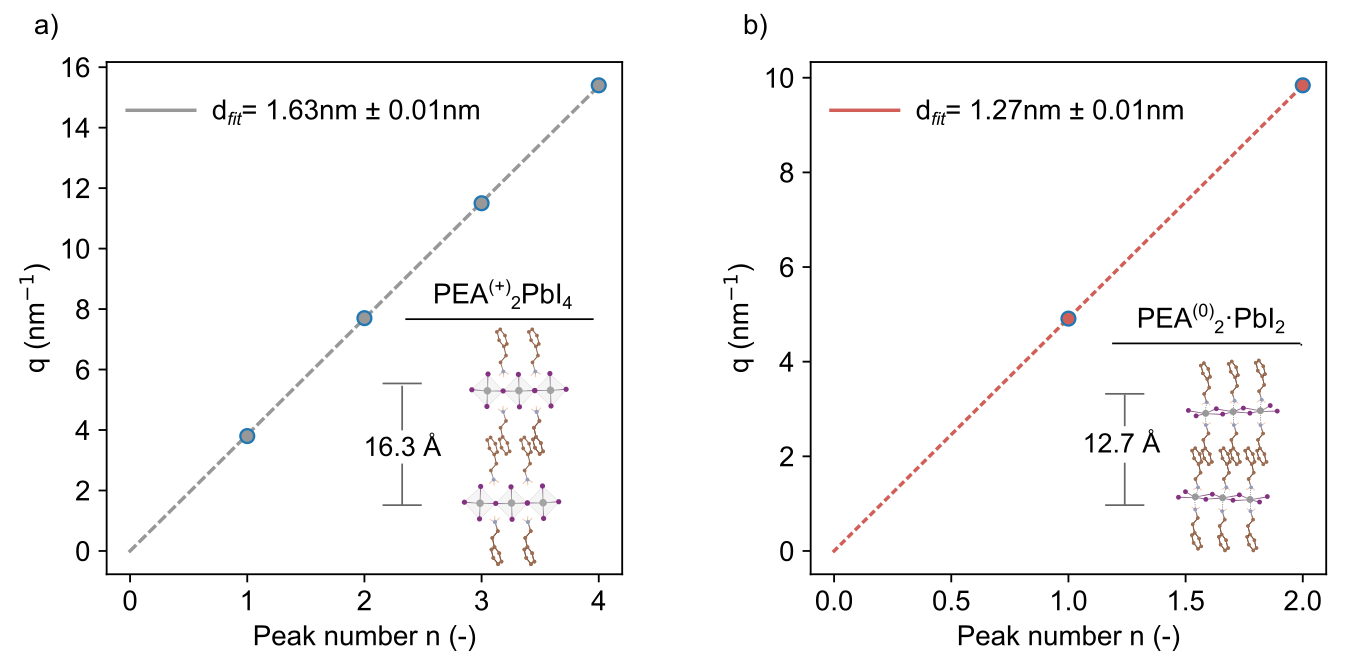


**Figure S2. Interplanar distance calculated from the slope of the reflection number against the q value.** a) linear fit for the pristine sample (PEA^(+)^_2_PbI_4_). b) linear fit for the intercalated structure PEA^(0)^_2_PbI_2._ The interplanar distance was calculated from the Bragg equation using a diffraction vector q, $d=\frac{2\pi}{slope}$ .


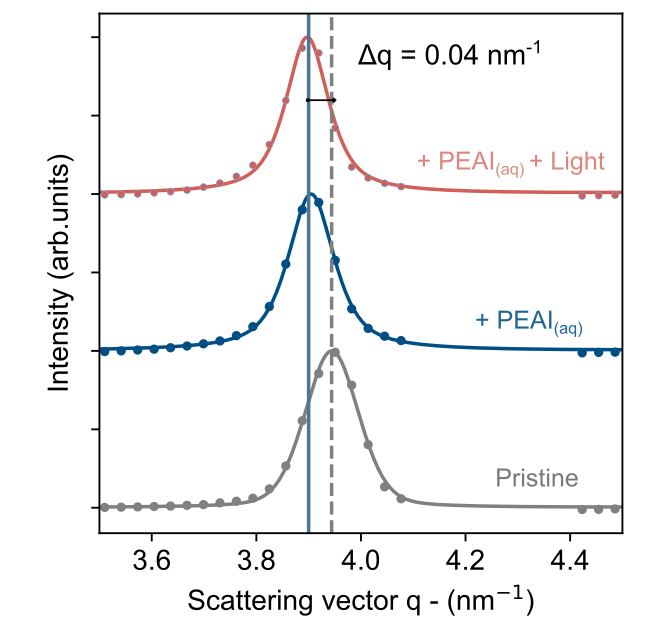


**Figure S3. Changes in the position of the diffraction peak at 002.** The figure shows a shift towards lower values of *q* after immersion in the electrolyte. To calculate the shift, the peak position was fitted with a pseudo-Voigt function.


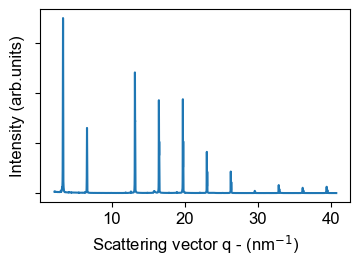


**Figure S4. X-ray diffraction spectra of films prepared using a PEAI solution.** The films were fabricated by drop-casting PEAI solution onto an ITO substrate and measured using a Cu source with Bragg-Brentano geometry. The values were converted to q values for easier comparison with the measured values in GIWAXS.


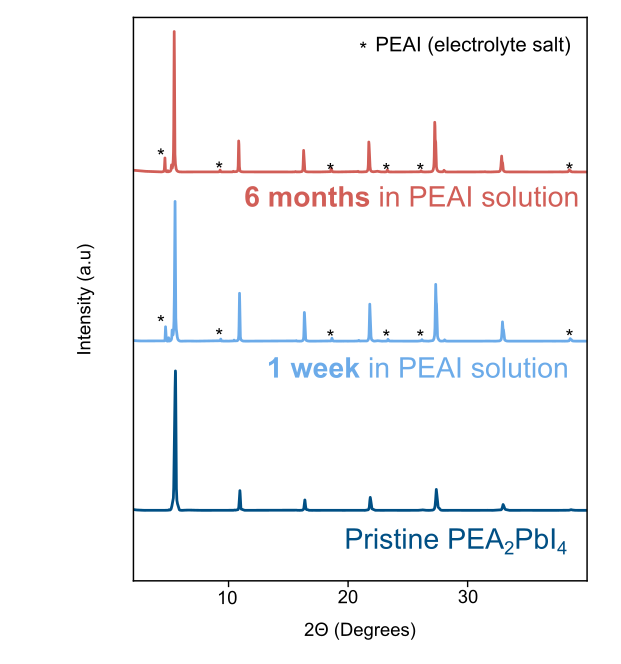


**Figure S5**. XRD diffractogram of the thin-film immersed in the electrolyte solution for long time. No changes in the crystal structure were observed after 6 months of exposure to the media.


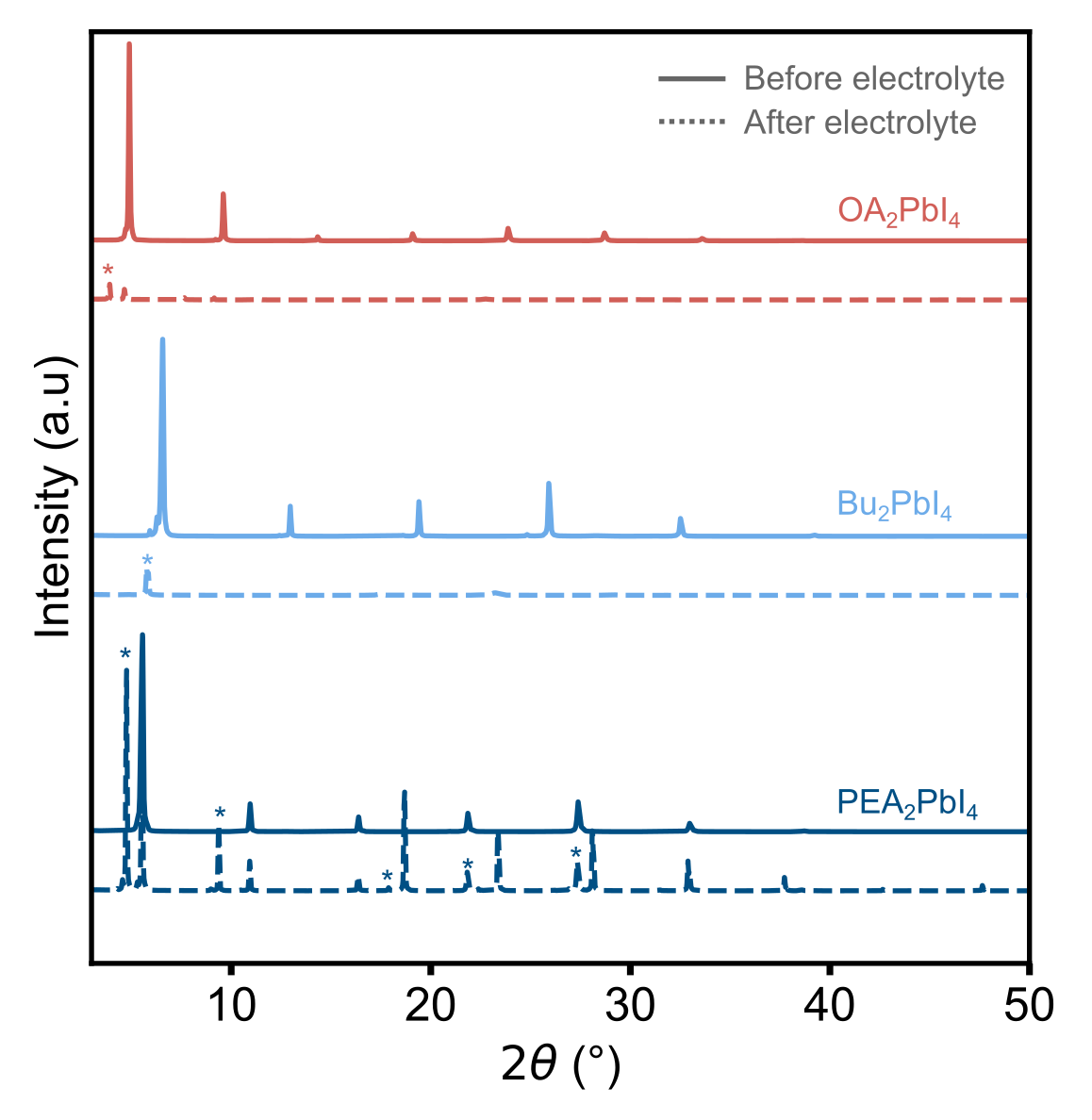


**Figure S6.** X-ray diffraction patterns for three different RP materials (OA₂PbI₄ – octylammonium lead iodide, Bu₂PbI₄ – butylammonium lead iodide, PEA₂PbI₄ – phenethylammonium lead iodide) before (solid line) and after (dotted line) immersion in electrolytes containing 1 M of OAI, BuI, and PEAI, respectively. The asterisk (*) denotes the diffraction peak associated with the respective cation salt observed after electrolyte precipitation.


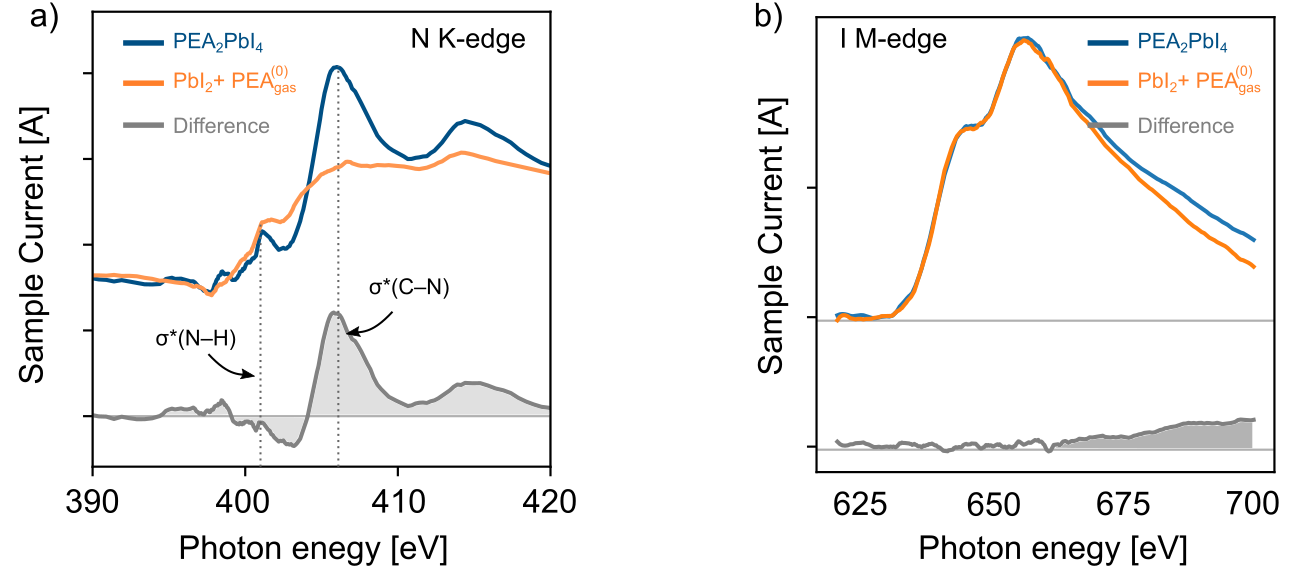


**Figure S7** Near-edge X-ray absorption fine structure (NEXAFS) spectra of the pristine and adduct 2PEA^(0)·^PbI₂ phase. (a) N K-edge spectrum, highlighting changes in the intensity of the C-N bond. (b) I M-edge spectrum.


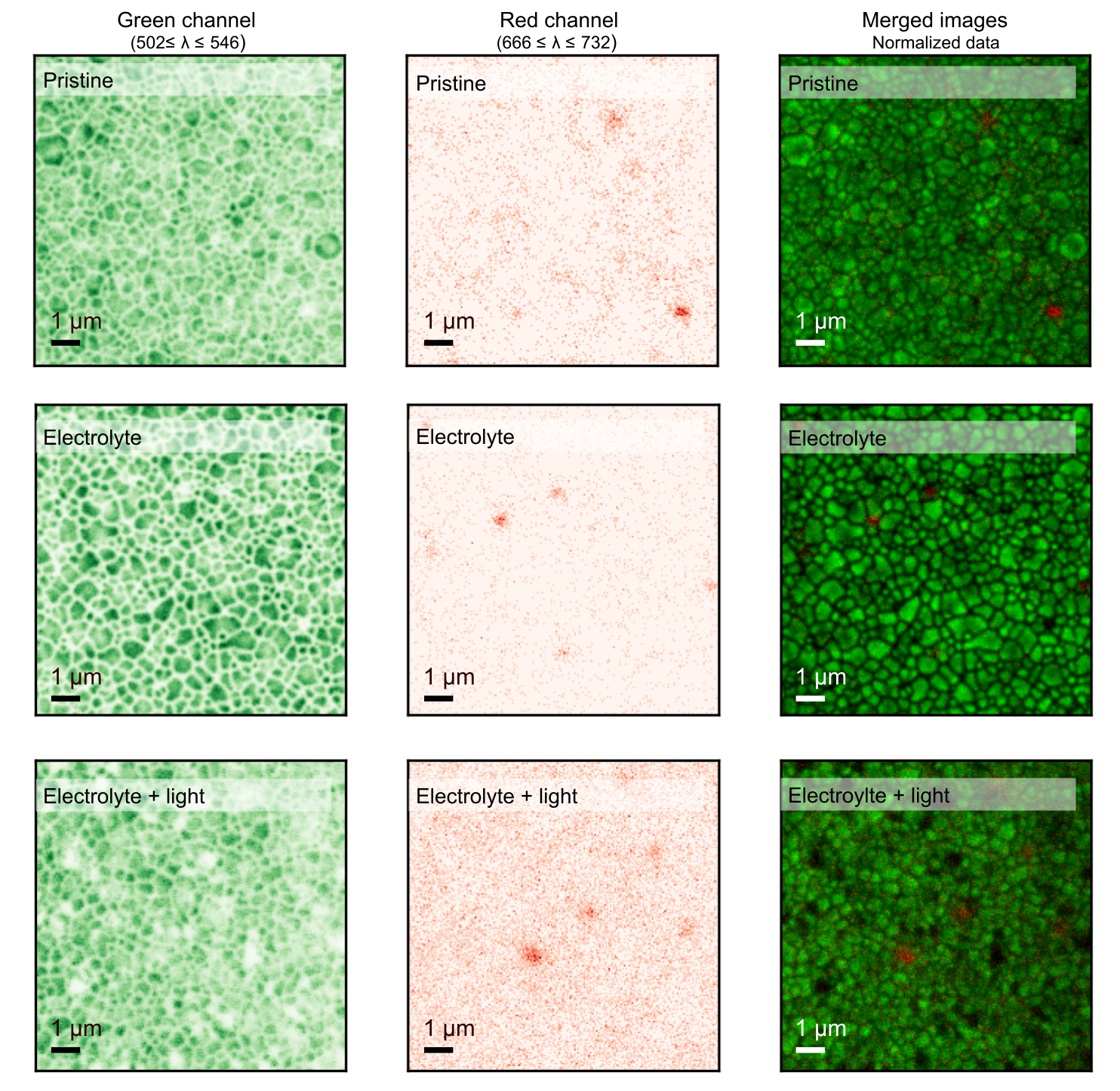


**Figure S8. Confocal laser scanning microscopy (CLSM) for the three conditions: Pristine, electrolyte, and electrolyte + light. The columns represent separation of emission in the different channels and their combination as indicated.**


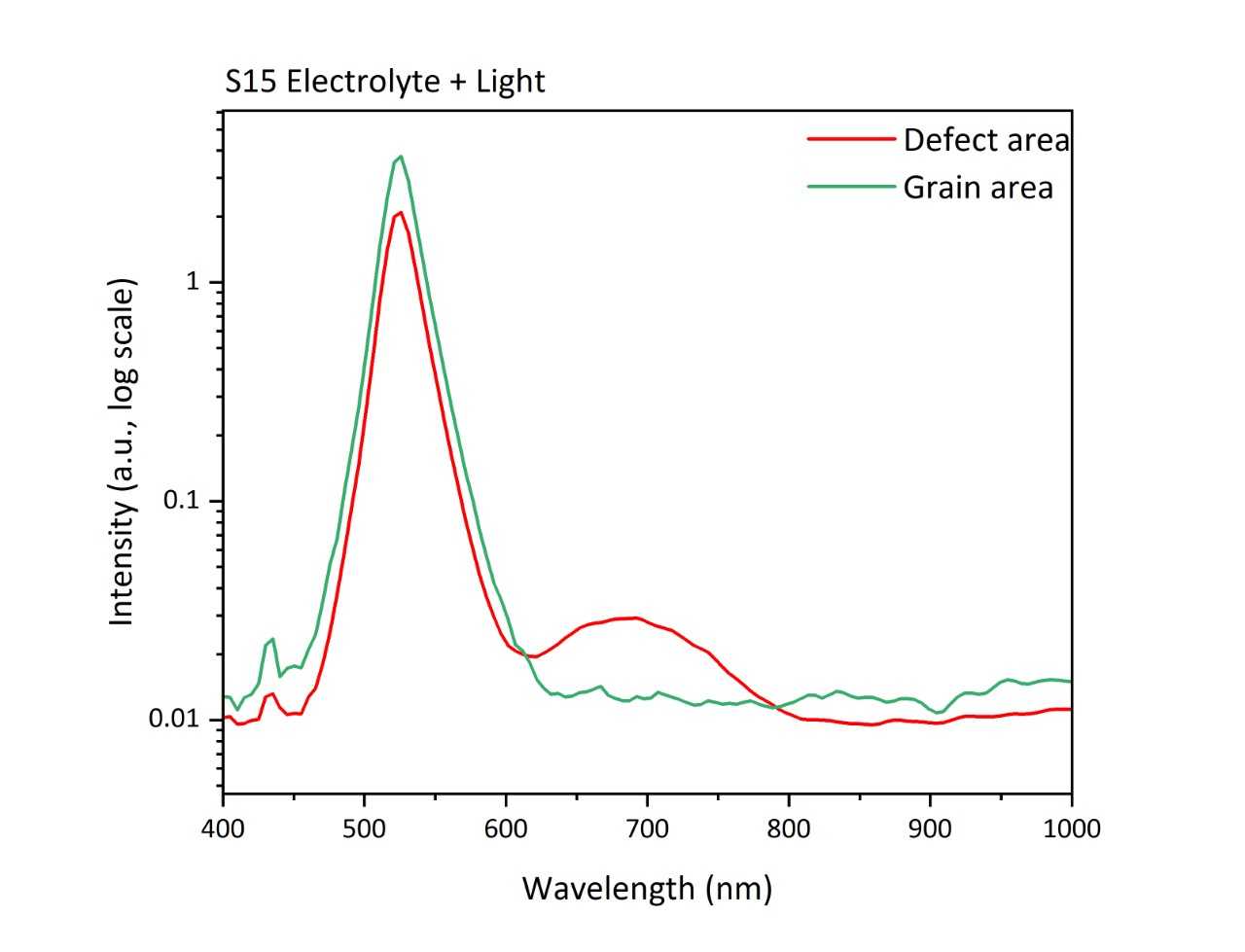


**Figure S9. Hyperspectral spectrum of the grain area and the defect area in a sample after being exposed to electrolyte and light. The broad emission in the sub-bandgap zone is associated with localized material defects.**


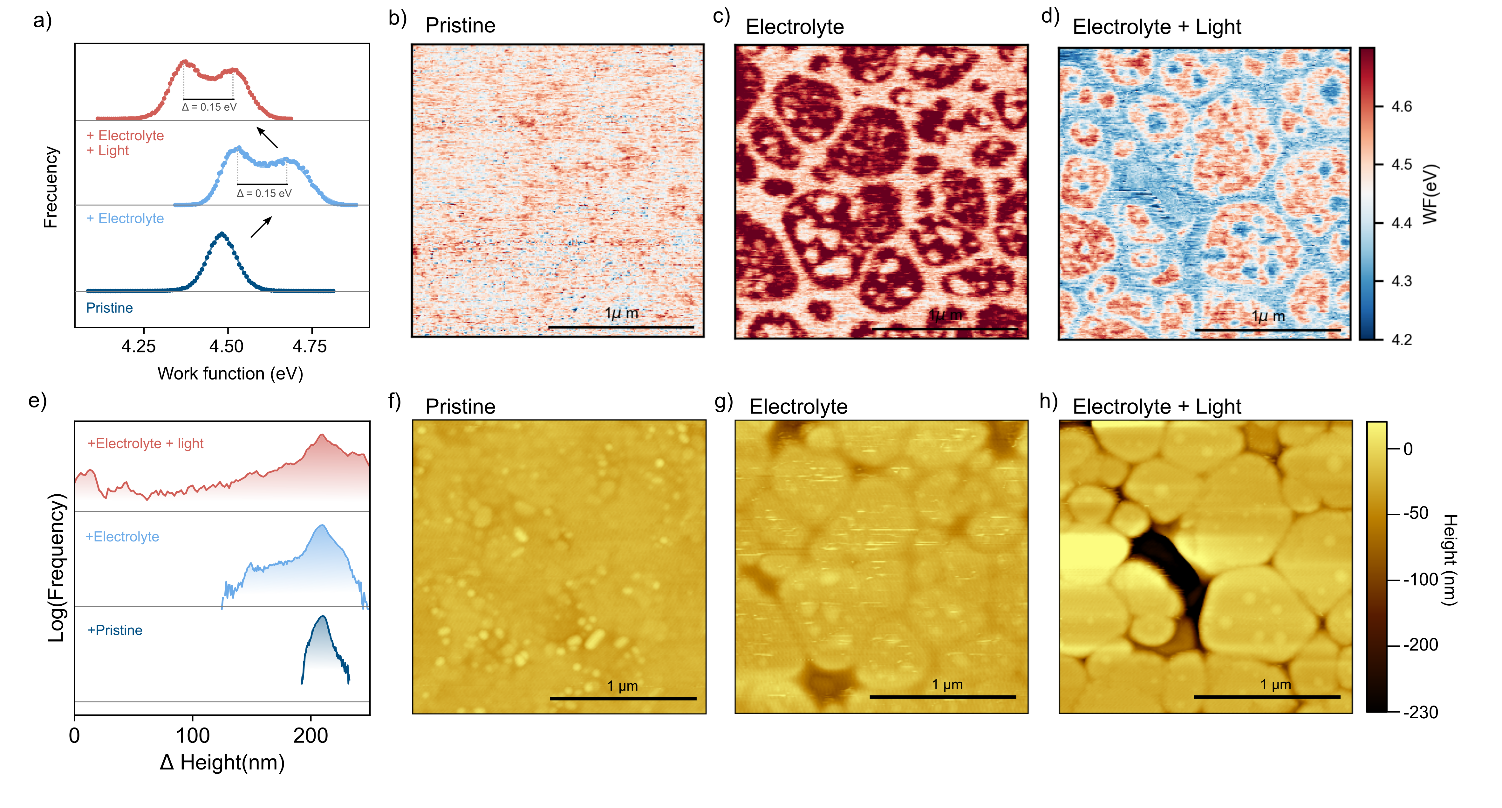


**Figure S10. Height change and work function change under the three experimental conditions measured by AFM and FM-KPFM.**


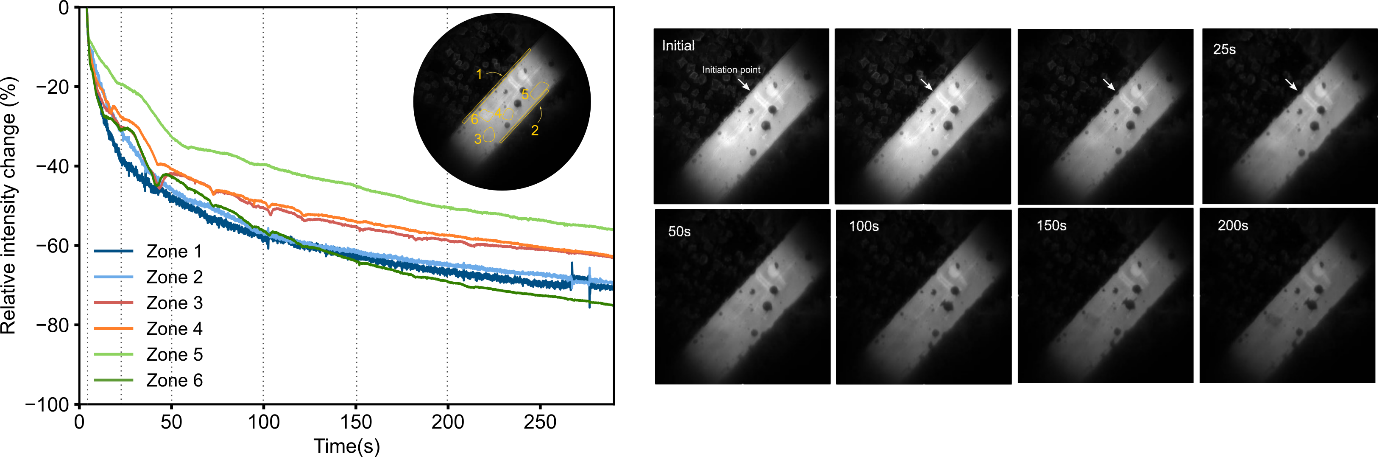


**Figure S11.** **Photodegradation of a PEA₂PbI₄ crystal under light irradiation monitored by photoluminescence (PL) imaging.** a) Relative change in PL intensity over time at selected regions of the crystal. Zones 1 and 2 correspond to the crystal edges, while the remaining zones are located within the interior. b) Time-resolved PL images showing the spatial evolution of the emission during continuous illumination. The measurements were performed under 415nm LED illumination.


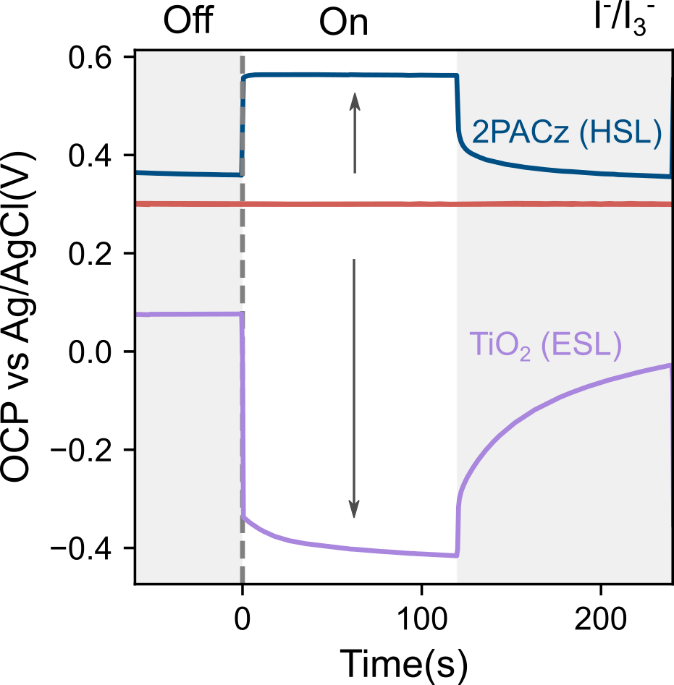


**Figure S12. Changes of open-circuit potential using a hole selective layer (HSL) and an electron-selective layer (ESL).** I^–^/I_3_^–^ was used as a redox probe to fix the redox potential in solution.


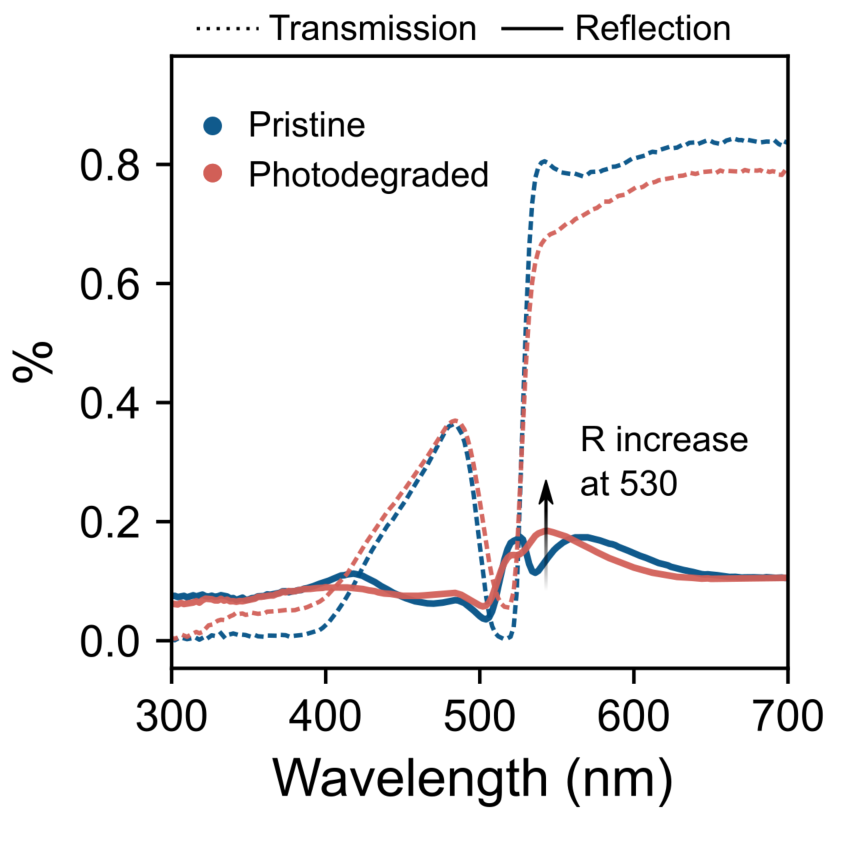


**Figure S13. Transmission and reflection data for a pristine sample and after degradation.** The increase in the reflection spectra below the band gap suggests that changes in the absorption of the sample below the bandgap are due to scattering.


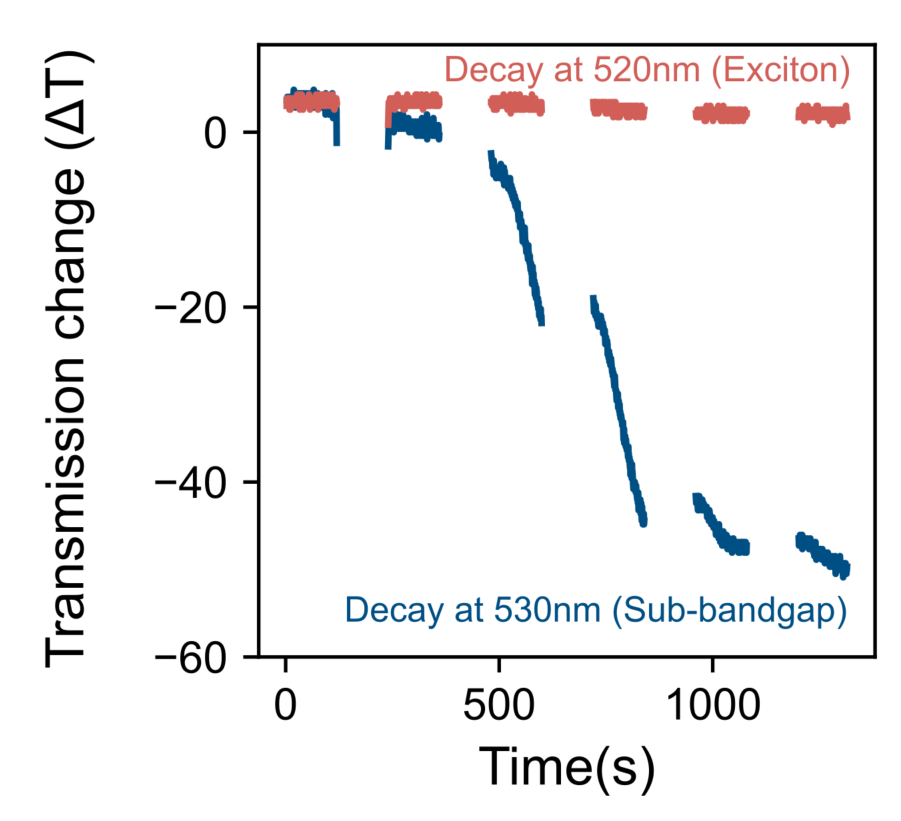


**Figure S14. Transmission change at the sample upon illumination without redox probe.** The absence of changes in the exciton absorption region contrasts with the dramatic reduction in the sub-bangap region.


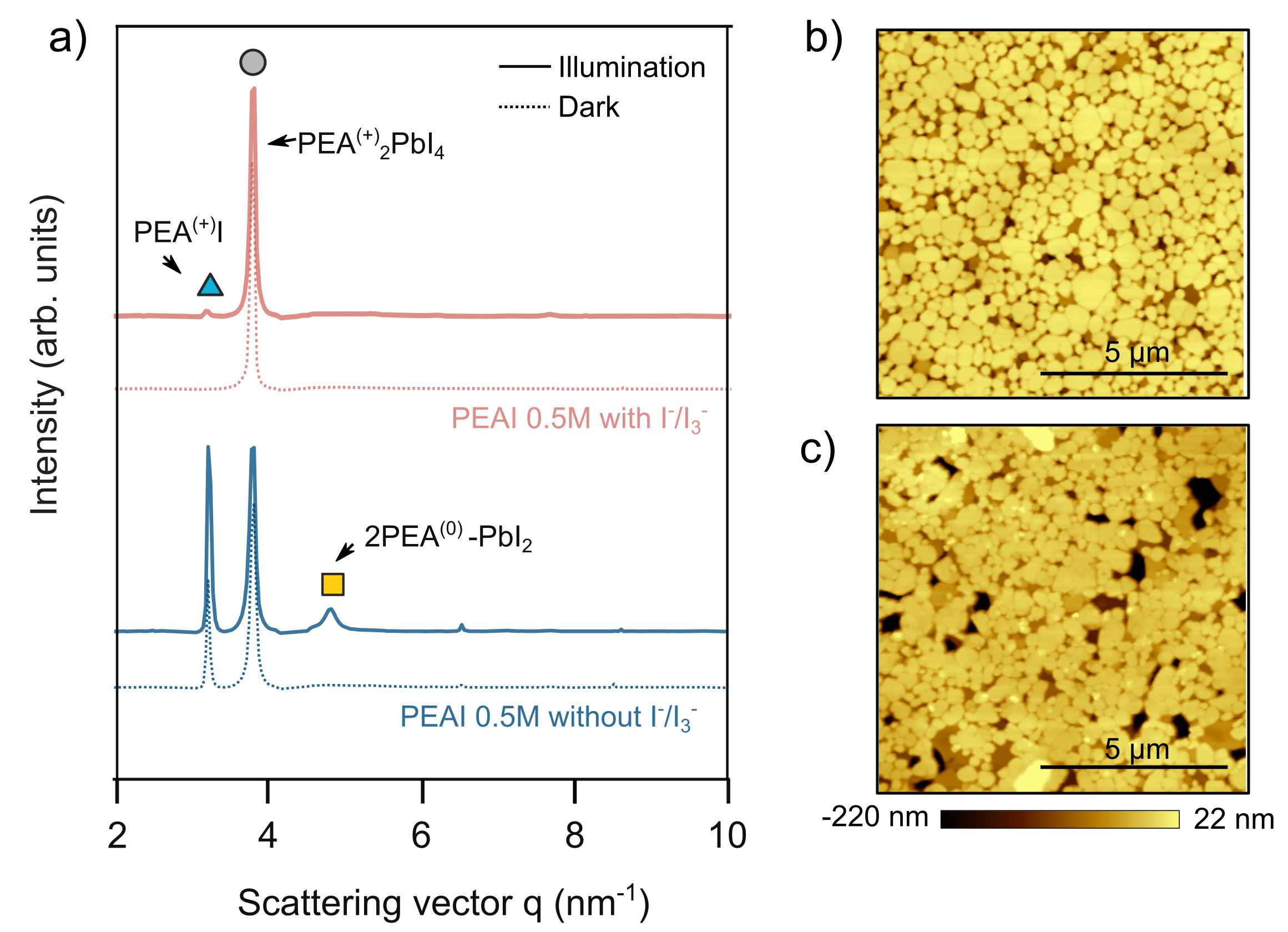


**Figure S15. Structural and morphological changes with the use of I^-^/I_3_^-^ as redox mediator.** a) Azimuthal integrated diffractogram with and without redox mediator, before and after illumination. The adduct formation is suppressed in the presence of the iodine mediator. **b)** AFM morphology for the case of I^-^/I_3_^-^ and c) AFM morphology for the case without redox mediator.


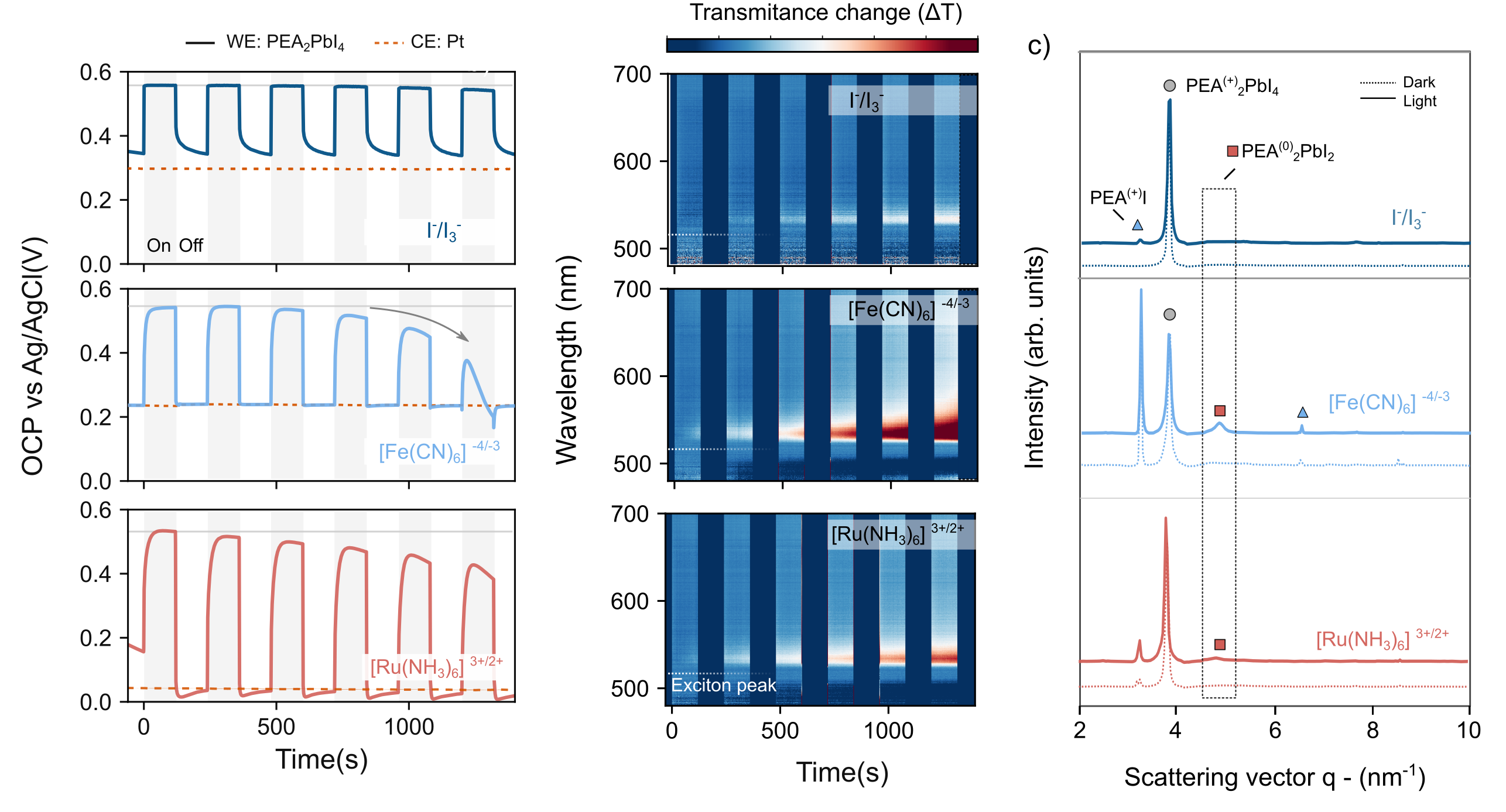


**Figure S16**: **Photodegradation Dynamics with Three Redox Mediators Under Chopped Illumination** (a) Open-circuit potential (OCP) measurements under chopped illumination with a 2-minute period for three redox mediators illustrating different degradation kinetics: I^-^/I_3_^-^ (Top – minimal degradation), [Fe^II/III^(CN)_6_]^4–/3–^ (middle – strong degradation), ([Ru^II/III^(NH_3_)_6_]^2+/3+^) (bottom – medium degradation). The solid lines show the potential of the PEA^(+)^_2_PbI_4_ photoelectrode, while the dotted lines represent the potential of the solution measured against the platinum counter-electrode. (b) In situ transmittance measurements (ΔT) during the OCP cycles indicate spectral changes over time for the three redox mediators. Changes are especially evident in the sub-bandgap spectral region, demonstrating the impact of photodegradation on the morphology and the attainable photovoltage. (c) Ex situ GIWAXS patterns recorded in the dark and after illumination using the redox mediators, illustrating the structural changes in the materials induced by the photodegradation process


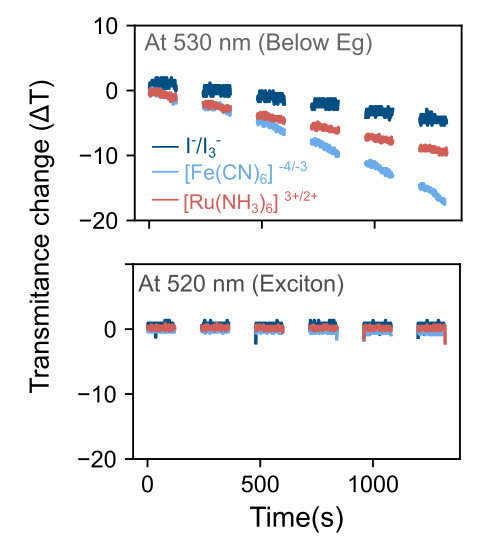


**Figure S17. Transmission change at the sample upon illumination with 3 redox probes,** I^−^/I_3_^−^ , [Fe^II/III^(CN)_6_]^4–/3–^, and the [Ru^II/III^(NH_3_)_6_]^2+/3+^, respectively. All the samples were measured using a concentration of 2mM in aqueous PEAI (125 mg/ml). The top graphic represents the changes in the sub-bangap region (530 nm) and the down graphic represent the changes at the exciton peak (520 nm). Notably no changes were observed at the exciton peak for the different redox probe conditions.


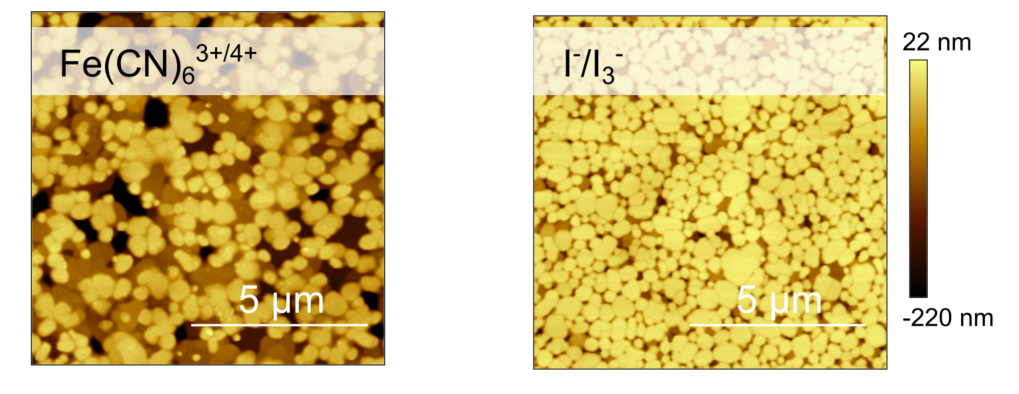


**Figure S18.** AFM images of PEA_2_PbI_4_ films exposed to electrolytes with [Fe^II/III^(CN)_6_]^4–/3–^and I^–^/I_3_^–^ redox couples.


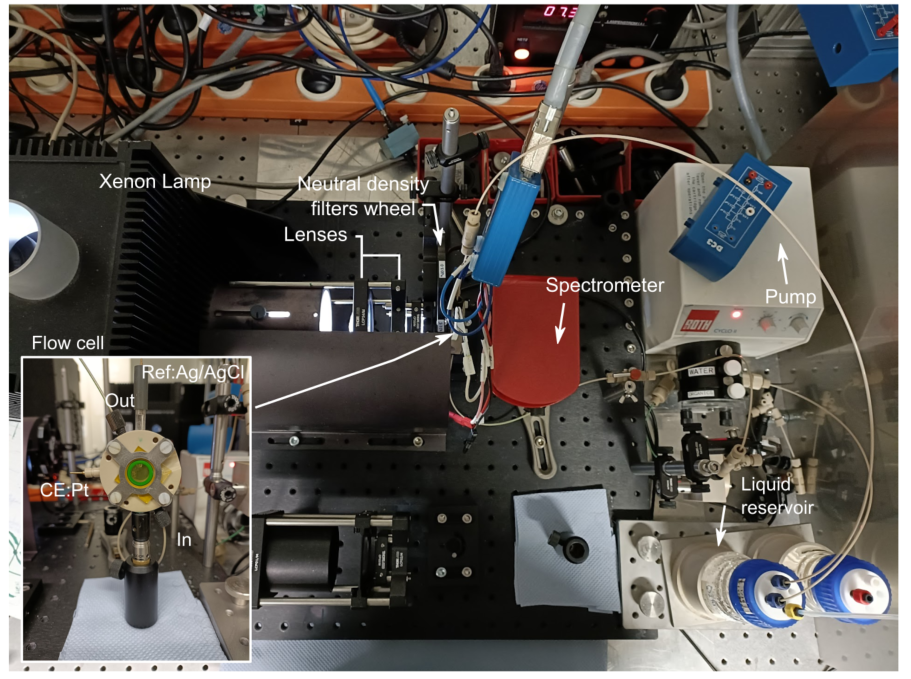


**Figure S19.** Photoelectrochemical station used in the current study.


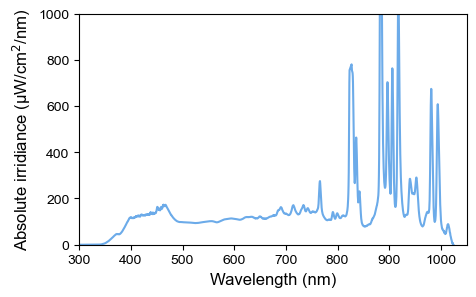


**Figure S20.** Irradiance spectrum of the xenon lamp with filter ND 0.5.

**References**

[1] T. W. Gries *et al.*, “Enhanced Electron Extraction in Co-Doped TiO2 Quantified by Drift-Diffusion Simulation for Stable CsPbI3 Solar Cells,” Mar. 2024. [Online]. Available: http://arxiv.org/abs/2403.11982

[2] Y. Liu *et al.*, “Multi-inch single-crystalline perovskite membrane for high-detectivity flexible photosensors,” *Nat. Commun.*, vol. 9, no. 1, pp. 1–11, 2018, doi: 10.1038/s41467-018-07440-2.

[3] G. Ashiotis *et al.*, “The fast azimuthal integration Python library : pyFAI,” *J. Appl. Crystallogr.*, vol. 48, pp. 510–519, 2015, doi: 10.1107/S1600576715004306.

[4] S. Banerjee, T. Hemraj-Benny, S. Sambasivan, D. A. Fischer, J. A. Misewich, and S. S. Wong, “Near-edge X-ray absorption fine structure investigations of order in carbon nanotube-based systems,” *J. Phys. Chem. B*, vol. 109, no. 17, pp. 8489–8495, 2005, doi: 10.1021/jp047408t.

[5] U. Zerweck, C. Loppacher, T. Otto, S. Grafström, and L. M. Eng, “Accuracy and resolution limits of Kelvin probe force microscopy,” *Phys. Rev. B*, vol. 71, p. 125424, 2005, doi: 10.1103/PhysRevB.71.125424.

[6] A. Axt, I. M. Hermes, V. W. Bergmann, N. Tausendpfund, and S. A. L. Weber, “Know your full potential : Quantitative Kelvin probe force microscopy on nanoscale electrical devices,” pp. 1809–1819, 2018, doi: 10.3762/bjnano.9.172.

[7] V. Online and E. Citation, “High-sensitivity quantitative Kelvin probe microscopy by noncontact ultra-high-vacuum atomic force microscopy,” *Appl. Phys. Lett.*, vol. 75, pp. 286–288, 1999, doi: 10.1063/1.124357.

[8] P. review B, “Angle-resolved ultraviolet photoelectron spectroscopy of the unoccupied band structure of graphite,” vol. 32, no. 12, 1985, doi: 10.1103/PhysRevB.32.8317.
